# Single 3′-exonuclease-based multifragment DNA assembly method (SENAX)

**DOI:** 10.1038/s41598-022-07878-x

**Published:** 2022-03-07

**Authors:** Viet Linh Dao, Sheena Chan, Jingyun Zhang, Russell Kai Jie Ngo, Chueh Loo Poh

**Affiliations:** 1 Department of Biomedical Engineering, National University of Singapore, Singapore, Singapore; 2 Synthetic Biology for Clinical and Technological Innovation, National University of Singapore, Singapore, Singapore

**Keywords:** Genetic engineering, Biological techniques, Synthetic biology

## Abstract

DNA assembly is a vital process in biotechnology and synthetic biology research, during which DNA plasmids are designed and constructed using bioparts to engineer microorganisms for a wide range of applications. Here, we present an enzymatic homology-based DNA assembly method, SENAX (Stellar ExoNuclease Assembly miX), that can efficiently assemble multiple DNA fragments at ambient temperature from 30 to 37 °C and requires homology overlap as short as 12–18 base pairs. SENAX relies only on a 3′–5′ exonuclease, XthA (ExoIII), followed by *Escherichia coli* transformation, enabling easy scaling up and optimization. Importantly, SENAX can efficiently assemble short fragments down to 70 bp into a vector, overcoming a key shortcoming of existing commonly used homology-based technologies. To the best of our knowledge, this has not been reported elsewhere using homology-based methods. This advantage leads us to develop a framework to perform DNA assembly in a more modular manner using reusable promoter-RBS short fragments, simplifying the construction process and reducing the cost of DNA synthesis. This approach enables commonly used short bioparts (e.g., promoter, RBS, insulator, terminator) to be reused by the direct assembly of these parts into intermediate constructs. SENAX represents a novel accurate, highly efficient, and automation-friendly DNA assembly method.

## Introduction

DNA assembly is an important and routine process in biotechnology and synthetic biology research, during which plasmids are designed and constructed using DNA parts to build genetic circuits to reprogram the cells. Several DNA assembly methods have been developed over the years, and the methods can be categorized based on the operating conditions (in vivo or in vitro)^1^. While in vivo assembly appears to be useful for the assembly of long DNA fragments, it still has low efficiency and is difficult to optimize. On the other hand, in vitro assembly methods have been widely employed for routine DNA construction, as they are more stable and have higher efficiency and accuracy. In in vitro methods relying on restriction enzymes (RE-based methods), the DNA parts are flanked by restriction sites that allow joining of multiple DNA fragments. Recently, reported RE-based assembly frameworks (BASIC; Golden Gate; MOBIUS) have enabled DNA assembly to be performed in a modular manner^2–4^. However, RE-based methods generally involve cycles of tedious digestion and ligation reactions and introduce unwanted scars into the constructs, and the joining fragments are required to be free of restriction sites used in assembly, complicating the design and assembly process. Sequence homology-based DNA assembly allows researchers to avoid these issues.

Cloning using in vitro homology-based methods (or sequence-overlapping methods) (e.g., Gibson assembly and In-Fusion assembly) has gained popularity because these methods enable seamless assembly of multiple fragments with high efficiency. These methods generally utilize a mixture of enzymes, including polymerases, exonucleases and ligases, in an isothermal process that relatively eases the whole protocol. Unlike RE-based methods, homology-based methods are sequence-independent, which simplifies the design for DNA assembly. However, in comparison with RE-based methods, homology-based assembly methods offer lower modularity^1^, limiting the reusability of parts for assembly. Many of the established modular DNA assembly methods are RE-based methods, particularly using the Type IIs RE and when the number of parts that need to be assembled is large^2–4^. On the other hand, the modularity of homology-based methods will require the design of predefined sets of synthetic linkers in which a number of aspects (e.g., orthogonality among the oligo sequences, GC content and distribution, and likelihood of forming hairpins) need to be taken into account^1^. In synthetic biology, well-characterized parts (such as promoter/insulator/reporter/terminator) are useful, and thus, modularization of these parts is highly desirable. Unfortunately, many of these parts are short and need to be linked together without flanking sequences. It is known that it is difficult to clone short DNA fragments directly using current homology-based methods^2,5^, such as by Gibson^6^ and SLIC^7^. In metabolic engineering and synthetic biology, it is commonly required to insert short fragments such as promoters/RBSs to tune the gene expression levels. To use homology-based methods for insertion of a short fragment, one approach is to design and generate the primer with the sequence that includes the desired short part and use the long primers for PCR amplification of the fragment of interest for DNA assembly. This approach limits the modularity/reusability of DNA parts for assembly, complicates the workflow and design, and increases the cost of oligo synthesis. An ad hoc approach is still largely taken. Consequently, there is a need to develop a homology-based DNA assembly method that allows shorter fragments to be assembled directly, enables higher modularity in the DNA assembly workflow with more reusability of parts, and simplifies the protocol, such as to reduce the temperature required.

In this paper, we studied exonuclease type III (the XthA enzyme) from *Escherichia coli Stellar* cells for DNA assembly and developed a multifragment DNA assembly method (SENAX) that uses the XthA enzyme alone. XthA is known as a multifunctional DNA repair enzyme that has important biological roles in DNA metabolism (phosphatase, exonuclease, AP endonuclease, RNase H activities)^8,9^. Its homologues were reported to have critical roles in DNA repair, DNA replication, and DNA recombinant systems of cells, including *E. coli*, *Bacillus subtilis*, *Pseudomonas*, and *Mycobacterium tuberculosis*^9–12^. Recently, an in vivo assembly technique (iVEC) using *E. coli* was reported to be dependent on a complex of gene activities, including *XthA*^13^. The unique properties of the XthA enzyme also enabled its application in the catalysis of nucleoprotein complexes and even for the sequencing analysis of short DNA fragments^14^. Conley et al. previously proposed that XthA in *E. coli *in vivo could help in the circularization of linear plasmids without homologies^15^, while very recently, Yang et al. reported that the in vivo cloning activity of *E. coli* requires both XthA and ExoX^16^. In the same study, Yang et al. also presented a minimal experiment using XthA for the circulation of a linear plasmid with a 24 bp homology arm. However, it is unclear whether XthA alone would be sufficient for in vitro multiple fragment DNA assembly, such as for the assembly of more than 2 DNA-fragments. Hence, we studied and characterized the efficiency of using XthA in vitro for the multiple fragments DNA assembly in this paper. Xia et al. recently described a T5 exonuclease (a 5′-exonuclease)-dependent DNA assembly (TEDA)^17^, which is considered a simpler version of Gibson assembly because it removes the use of Phusion DNA polymerase and DNA ligase in the mix. In this study, while the reaction conditions were optimized for 2-fragment DNA assembly (cloning of a single gene fragment GFP into a single backbone), little is known about multifragment DNA assembly by TEDA, as the authors reported only an example of a 4-fragment assembly. Interestingly, in our study, we discovered that it is possible to achieve high efficiency in assembling multiple (up to 6) fragments in different example backbones using only XthA in a mix at ambient temperatures of 30–37 °C. The efficiency achieved by SENAX is comparable to that achieved by commonly used DNA assembly methods (Gibson and In-Fusion) while requiring shorter homology arms and lower reaction temperatures. More importantly, we found that we were able to effectively and directly insert a short fragment of DNA down to 70 bp into a medium-sized template backbone (1–10 kb) using only the XthA enzyme in the mix. To the best of our knowledge, this has not been reported elsewhere using homology-based assembly methods. Furthermore, taking advantage of the short fragment assembly capability, we developed a library of standard well-defined reusable DNA short bioparts, ranging from 70 to 100 bp. The library comprises a set of commonly used constitutive promoters and RBSs and can be easily reused for the construction of variants. Taken together, SENAX allows short homology arm design, enables direct short fragment assembly using a homology-based method, improves modularity, is easy to use, and requires low-energy consumption.

## Results

### Purified XthA from Stellar cell extract is sufficient for general DNA assembly

**Figure 1:**
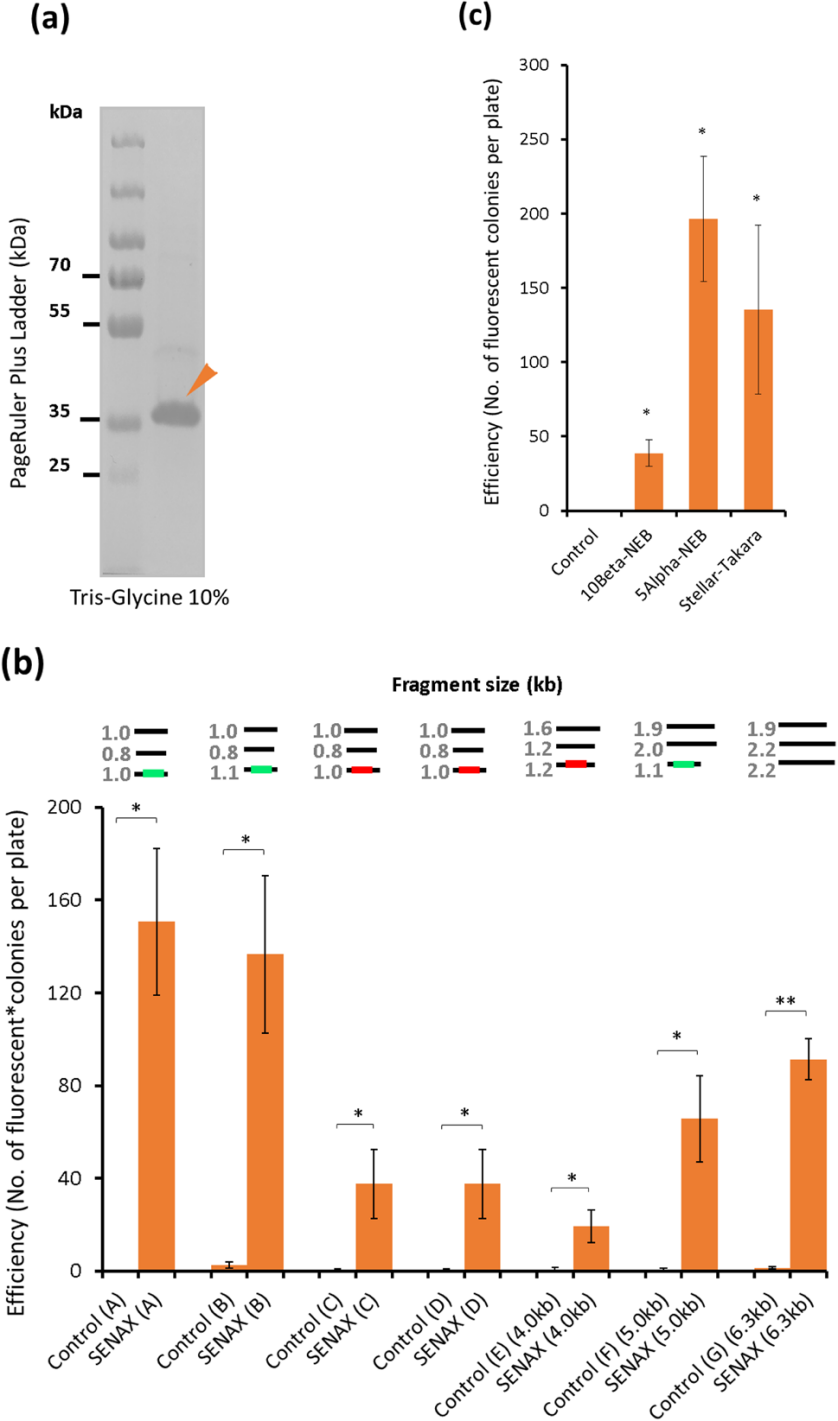
Purified XthA is sufficient for DNA assembly. (**a**) Purified XthA proteins were verified by 10.0% SDS PAGE. Lane 1 (left), protein marker; Lane 2, Purified XthA. (**b**) Efficiency of 3-fragment assembly by SENAX. Constructs A, B, C, D (2.8 kb); E (4.0 kb); F (5.0 kb); and G (6.3 kb) were used for the test (see Fig. S1 for details about the configuration of each construct). The size of the fragments used for assembly are presented on top of the plot (**b**): the red line represents RFP, and the green line represents GFP. The error bars represent the standard deviations (STDEVs) of three replicates. *p < 0.05, **p < 0.01 by paired t test against the control. Samples with no enzyme protein XthA were used as the control. (**c**) Transformation of plasmids (Construct B) assembled by SENAX using different competent cells, i.e., 10Beta (NEB), 5Alpha (NEB), and Stellar (Takara). The configurations of a replication origin (15A), antibiotic resistance (AmpR), and a green fluorescence gene were used for assembly. The obtained fluorescent colonies represented the efficiency of the method. The error bars represent the standard deviations (STDEVs) of three distinct replicates. *p < 0.05 by paired t test against the control. Samples with no enzyme protein were used as the control.

Motohashi previously reported that DNA assembly can be performed using *E. coli* cell extract^18^, a method named SLiCE assembly^19^. While a number of enzymes could be responsible for SLiCE assembly and in vivo recombination activity in *E. coli*^20^, recent reports revealed the important role of XthA and its homologues in DNA repair in many species, including *E. coli,* and XthA is required for in vivo DNA cloning using *E. coli*^13^. Hence, we hypothesized that XthA could be the reason behind the DNA cloning activity (SLiCE) of *E. coli* and could have a role in in vitro DNA assembly. We sought to study XthA to determine whether this enzyme has innate activity on DNA assembly. Although the XthA product is commercially available for molecular biology studies (e.g., NEB M0206 L), the storage buffer contains some chemicals that might affect DNA assembly. As a result, we initiated our study by producing and purifying the enzyme XthA to better confirm its activity on DNA assembly. To produce *E. coli Stellar* XthA for the study, XthA was cloned and expressed in *E. coli* BL21 (Fig. 1a). The expressed XthA was then purified for subsequent studies. The identity of the purified protein was confirmed by matrix-assisted laser desorption ionization-time of flight mass spectrometry (MALDI-TOF MS) (Fig. S2).

We first studied whether the enzyme XthA alone could assemble a small number of DNA fragments that would express green fluorescent protein (GFP) in vivo when assembled correctly (Fig. S3). To this end, we studied the efficiency of the mix with only XthA in assembling 3 typical medium-size fragments—an RSF origin of replication, a kanamycin resistance cassette, and a GFP reporter gene, which were PCR-prepared to have 18 bp overlapping regions to each other. Twenty nanograms of each fragment was used, and the reaction was performed at 37 °C for 15 min. Stellar cell extract, Stellar cell extract supplemented with XthA, and a mix without XthA were used as controls. From the results, we observed that the concentrated cell extract from *E. coli Stellar* had innate activity to assemble DNA parts as an obvious number of green fluorescent colonies grew on the screening plate (Fig. S3). This concurs with previously reported studies reporting SLiCE^18,19^. Interestingly, when only purified XthA was used for assembly, a much higher number of fluorescent colonies was obtained (Fig. S3). This result indicates that having only XthA was sufficient for DNA assembly. However, the efficiency was lower when the concentrated Stellar cell extract supplemented with XthA was used. These results suggest that the cell extract probably contained some competitors to XthA, such as other dominant exonucleases in *E. coli* (RecBCD) that could inhibit the activity of XthA^10,13,20^. For the sample that used only XthA, approximately 95% of the total colonies were fluorescent. To confirm the sequences, three colonies among these fluorescent colonies were examined using sequencing. All the colonies sent for sequencing had the correct sequences, suggesting that XthA can achieve high accuracy in DNA assembly. The samples with the same amount of DNA fragments but without enzyme XthA added had no colonies on the screening plate, indicating that the in vivo assembly is not effective.

### SENAX enables 3-fragment assembly

We further investigated the efficiency of SENAX for 3-fragment assemblies to produce a series of plasmids of varying sizes (2.8 kb-A/B/C/D; 4.0 kb-E, 5.0 kb-F; 6.3 kb-G) and with different bioparts, including different origins of replication and genes of interest (Figs. 1b and S1). The efficiency of assembly was evaluated based on either the number of fluorescent colonies on the antibiotic plate (all constructs) or the number of colonies grown on the plate (construct G). The results show that high efficiency was achieved as many fluorescent colonies appeared on the antibiotic plates (Fig. 1b). However, the number varied by template, ranging from 20 to 150. Nonetheless, the results demonstrated that SENAX is able to assemble common-size DNA fragments to generate a range of plasmid sizes.

Since XthA is expressed using the gene derived from Stellar *E. coli*, we sought to examine whether its activity is dependent on specific components of Stellar *E. coli*. Hence, we performed the 3-fragment assembly of construct B using different competent cells, including DH5-alpha and 10Beta (NEB), for the transformation step. The results show that DNA assembly activity based on the use of XthA was present even when different types of competent cells were used, although the cloning efficiency differed among the competent cells used (Fig. 1c). This suggests that the DNA assembly activity does not depend on specific components of Stellar *E. coli*. Taken together, the results demonstrated that XthA alone is sufficient for DNA assembly, as other enzymes (e.g., polymerase and ligases) are not present in the mix.

### SENAX can assemble up to 6 DNA fragments

**Figure 2:**
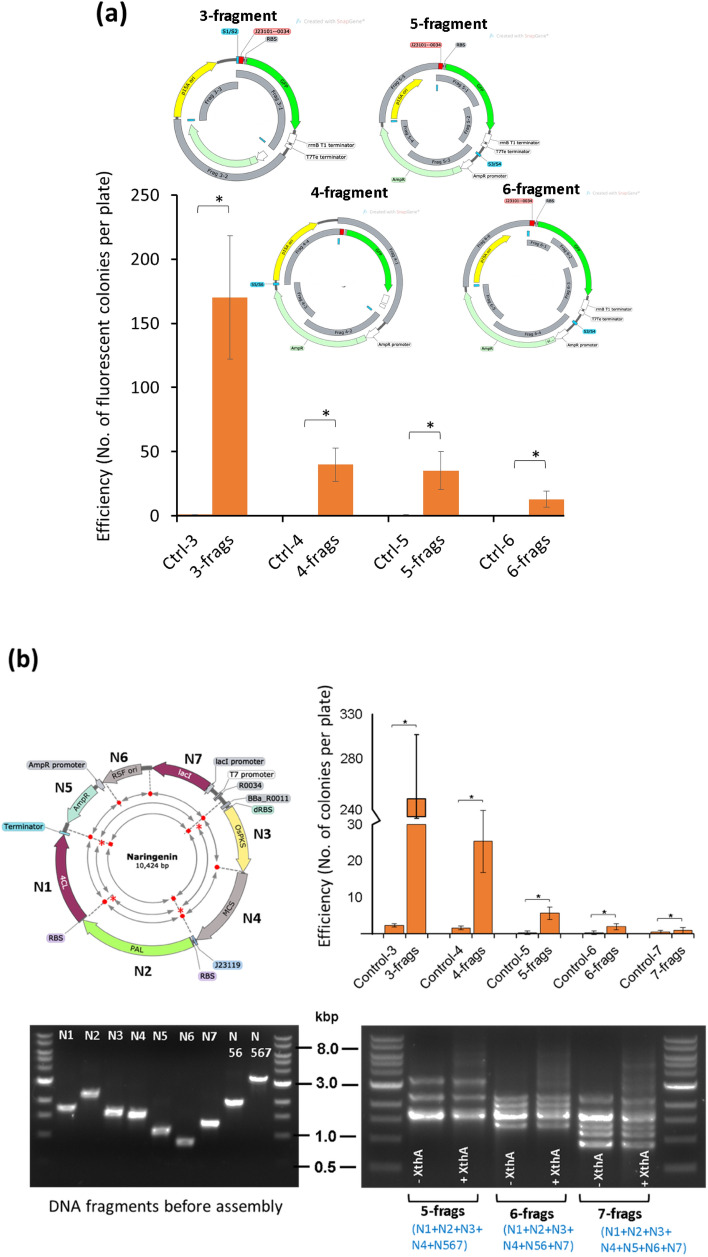
SENAX was tested with different numbers of DNA fragments. (**a**) A reporter plasmid (15A + AmpR + GFP) was separated by PCR into several linear fragments (3–4–5–6) with 18 bp homology. Top of the plot, an illustration of the configurations used in assembly tests. Fragments coloured in grey are the fragments used for assembly. The plot shows the efficiency of the assembly tests with an increasing number of fragments involved. (**b**) A naringenin-producing plasmid (10.5 kb) was separated by PCR into several linear fragments (3–4–5–6–7) with 18 bp homology arms. The fragments were then used for assembly reaction. Top left, an illustration of the configurations used in the assembly tests. Top right, a plot showing the efficiency of assembly along with an increasing number of fragments involved. Bottom left, DNA fragments with homology arms prepared by PCR verified by agarose gel electrophoresis. Bottom right, assembly mixes after incubation were verified by agarose gel electrophoresis, where the arrows indicate the expected intermediate assembly products. The error bars represent the standard deviations (STDEVs) of three distinct replicates. *p < 0.05, **p < 0.01 by paired t test against the controls. Control samples were prepared with the same amount of input DNA fragments but without enzyme protein XthA.

To evaluate the performance of SENAX in assembling multiple fragments, we performed assembly using constructs of varying sizes (2.8 kb and 10 kb) (Figs. 1b, 2) and with a varying number of joining fragments (3, 4, 5, 6 and 7) (Fig. 2). As presented in Fig. 2a, SENAX effectively catalyzed the assembly of 3, 4, or 5 fragments. SENAX was also able to assemble 6 fragments as a dozen fluorescent colonies were obtained. This number of fluorescent colonies of the 6-fragment assembly is approximately 90% less than that of the 3-fragment assembly and 70% less than that of the 4- or 5-fragment assembly. There were no colonies on the control plates, which were prepared by using the same amount of corresponding DNA fragments without supplementation with XthA enzyme.

We then investigated the multifragment assembly using a larger plasmid construct (10.5 kb) to gain further insight into the ability and limitation of SENAX (Fig. 2b). A gene cluster for naringenin synthesis under the control of constitutive promoters was cloned into the RSFori/AmpR backbone, resulting in a 10.5 kb plasmid (Fig. 2b). The plasmid was separated into 3, 4, 5 or 6 fragments using PCR, and these fragments were treated with DpnI to remove the circular template. The control samples were prepared by using a similar amount of input DNA without supplementation with the XthA enzyme. The negative colonies were mainly from the undigested vector, which was used as the PCR template but was incompletely digested by DpnI. Several hundred colonies were obtained from the plate of 3-fragment assembly, and the results revealed that the efficiency of assembly decreased exponentially with an increasing number of DNA fragments involved. This is a common observation, as reported for other assembly methods^2^. For the 6-fragment assembly, we obtained several colonies on the plate. Three colonies on each plate were picked and positively confirmed by colony PCR. Although a few colonies were observed with the 7-fragment assembly, the result was not consistent between the batches. Meanwhile, the background of the negative colonies, which possibly included the incorrect assembly, undigested template, and the potential assembly created in vivo*,* remained relatively constant. Overall, it was demonstrated that SENAX can handle DNA assembly in up to 6 DNA fragments well.

### Short-fragment (< 200 bp) assembly by SENAX

**Figure 3:**
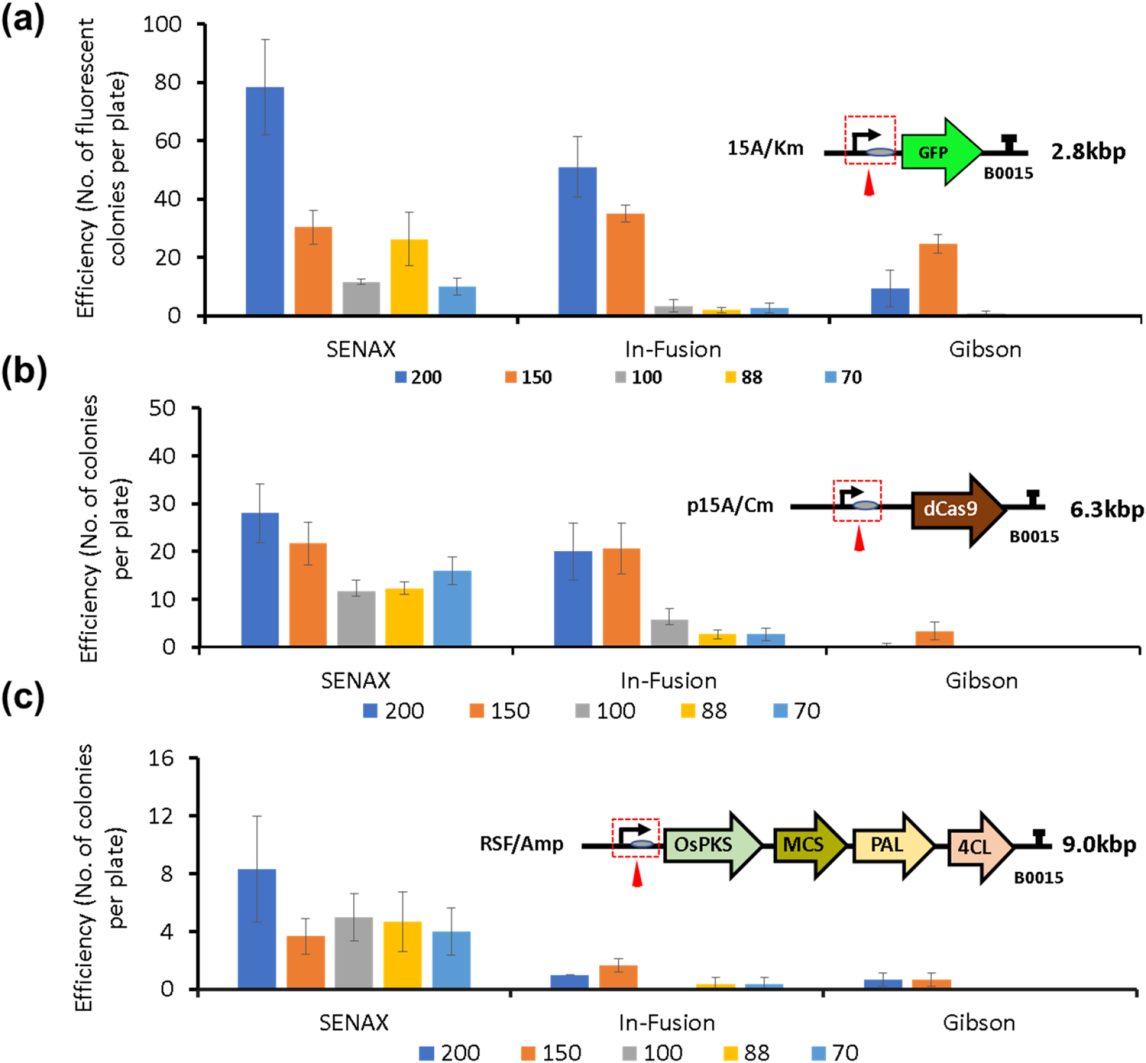
Short-fragment assembly by SENAX in comparison with commercial DNA assembly enzyme mixes. Short fragments with different lengths (200–150–100–88–70 bp) were introduced into variants of backbone templates by SENAX, In-Fusion (Takara), and Gibson (NEB). Do note that the two commercial kits were used according to the manufacturer recommended protocol and optimal conditions (e.g., at a temperature of 50 °C for at least 15 min). (**a**) Short fragments were introduced to the GFP reporter plasmid (2.8 kb). (**b**) Short fragments were introduced to the dCas9 expression plasmid (6.3 kb), and (**c**) short fragments were introduced to a naringenin-producing plasmid (9.0 kb). The error bars represent the standard deviations (STDEVs) of three distinct replicates.

To test the ability of SENAX to perform short fragment assembly, a library of short fragments (size varied—200 bp, 150 bp, 100 bp, 88 bp, 70 bp) made up of a specific set of promoter and RBS pairs was assembled with different template linear plasmids (backbone) and transformed into *E. coli*. The promoters and RBS were selected from the Anderson collection^21^. We sought to investigate the influence of the size of the short fragment and the size of the backbone on the efficiency of the short-fragment DNA assembly. To this end, five short fragments of different lengths (200 bp, 150 bp, 100 bp, 88 bp, 70 bp) were designed (Table S2). All of the short fragments consist of an 18 bp-specific spacer at the 5′ terminus, a promoter, and an RBS. The capability and efficiency of assembling the short fragments into variants of the backbone template of different lengths (2.8 kb, 6.8 kb, and 9.0 kb) were studied (Fig. 3). The results show that the short fragments were successfully inserted in the upstream region of either a GFP reporter gene (Fig. 3a) or other genes of interest, including the *dCas9* gene (Fig. 3b) and naringenin-producing gene cluster (Fig. 3c). The number of colonies obtained on the screening plate varies among the templates and decreases with increasing backbone template size. Two popular DNA assembly enzyme mixes (Gibson and In-Fusion) were also used in the experiment to evaluate the efficiency with SENAX. It should be noted that the kits were used according to the manufacturer's protocol to ensure that the kits were being used under their respective optimal conditions. With the 2.8 kb backbone plasmid, both Gibson and In-Fusion methods generated a number of fluorescent colonies for the assembly of the 200 bp and 150 bp short fragments. The efficiency of SENAX was comparable to that of In-Fusion, and both were higher than that of Gibson. However, In-Fusion and Gibson were rarely able to generate colonies with the insertion of short fragments of 100 bp, 88 bp, or 70 bp in size. Similar results for fragments shorter than 100 bp were obtained in the cases of 6.3 kb and 9.0 kb backbones for In-Fusion and Gibson. Both methods were not effective with fragments shorter than 100 bp, particularly with the 9.0 kb backbone. For these larger backbones, In-Fusion remained effective with the assembly of 200 bp and 150 bp fragments, while Gibson did not. In contrast, SENAX could handle short fragments from 200 bp down to 70 bp, as colonies with short fragments inserted were obtained for the three backbones tested, although the number of colonies with the largest backbone was not high. Based on the assembly of short fragments using the 6.3 kb and 9.0 kb backbones, PCR was used to verify whether the grown colonies correctly harbour the short-fragment insert (Fig. S4). The results showed that 11/12 (91.7%) of the selected colonies from the 200 bp and 150 bp samples were correct. Meanwhile, 8/12 (66.7%) of the selected colonies from the 100–88 bp samples were correct, and 8/14 (57.1%) of the selected colonies from the 70 bp samples were correct. The results show that SENAX is much more effective in assembling fragments shorter than 100 bp into backbones of varied sizes compared to Gibson and In-Fusion.

Among the tested short fragment sizes, 88 bp appeared to be a good candidate size to harbour bioparts such as promoters and RBSs, which are routinely used for fine-tuning gene expression. Within this fragment, we can incorporate a unique spacer, the full sequence of an Anderson constitutive promoter, a short spacer between promoter and RBS, and a common size RBS. To take advantage of SENAX’s ability to assemble short fragments directly, we created a library of 88 bp fragments comprising promoters of varying strength (Bba_23119, Bba_J23100, Bba_J23101, Bba_J23106) layered with an RBS (RBS0034) (see supplementary Table S2), which can be reused using SENAX to be assembled into different backbone templates (Fig. S5a–g). Based on the sequencing results that we obtained (a total of 18 colonies from 7 plates), an average success rate of 88.9% per plate was achieved (see Fig. S5—Table S3). This further shows that 88 bp fragment assembly is reliable and has the potential to be used as a standardized assembly framework. On the other hand, as we sought to determine the limitation in the size of the short fragment that can be assembled by SENAX, we performed an additional test with the 70 bp fragment and a 60 bp fragment using the *ho1* template (3.0 kb) (Fig. S5h). Tests of the 70 bp fragment with the *ho1* template were successful, achieving a high number of colonies (36). However, no colony was obtained with the 60 bp fragment assembly, suggesting that 70 bp would most likely be the limit. To verify that the 70 bp fragment was inserted correctly in the colonies that grew on the screening plate, 3 colonies from each plate were sent for sequencing verification (Fig. S5h). The sequencing results show that all of the sequenced colonies (12) were correct, suggesting that high accuracy was achieved. This implies that the enzyme XthA has precise activity to catalyze the correct short-fragment assembly. Taken together, SENAX can achieve high accuracy at reasonable efficiency for short-fragment assembly, and the minimum length of the short fragment that can be assembled directly into a template is 70 bp.

To further test SENAX assembly capability, we also used SENAX successfully to create a small combinatorial library of naringenin-producing plasmids (Fig. S6). While the different plasmids vary in promoter/RBS driving the respective GOIs, each plasmid consists of multiple repeated regions, including terminators, promoters, RBS, and spacers near the junctions, making assembly challenging. Nonetheless, correct constructs were obtained with reasonable accuracy, although efficiency was not high (data not shown).

XthA is also commercially available for research use, such as for the preparation of single-stranded DNA for dideoxy sequencing or site direct mutagenesis. A test using the commercial product (M0206 L—NEB) (with 30 times dilution) for 3-fragment assembly generated a number of fluorescent colonies (Fig. S7), although the number of colonies was lower compared with SENAX in the parallel experiment. This could be due to chemicals present in the storage buffer by the manufacturer.

### Optimization of the SENAX assembly reaction

#### Effect of XthA amount on the in vitro DNA assembly

**Figure 4:**
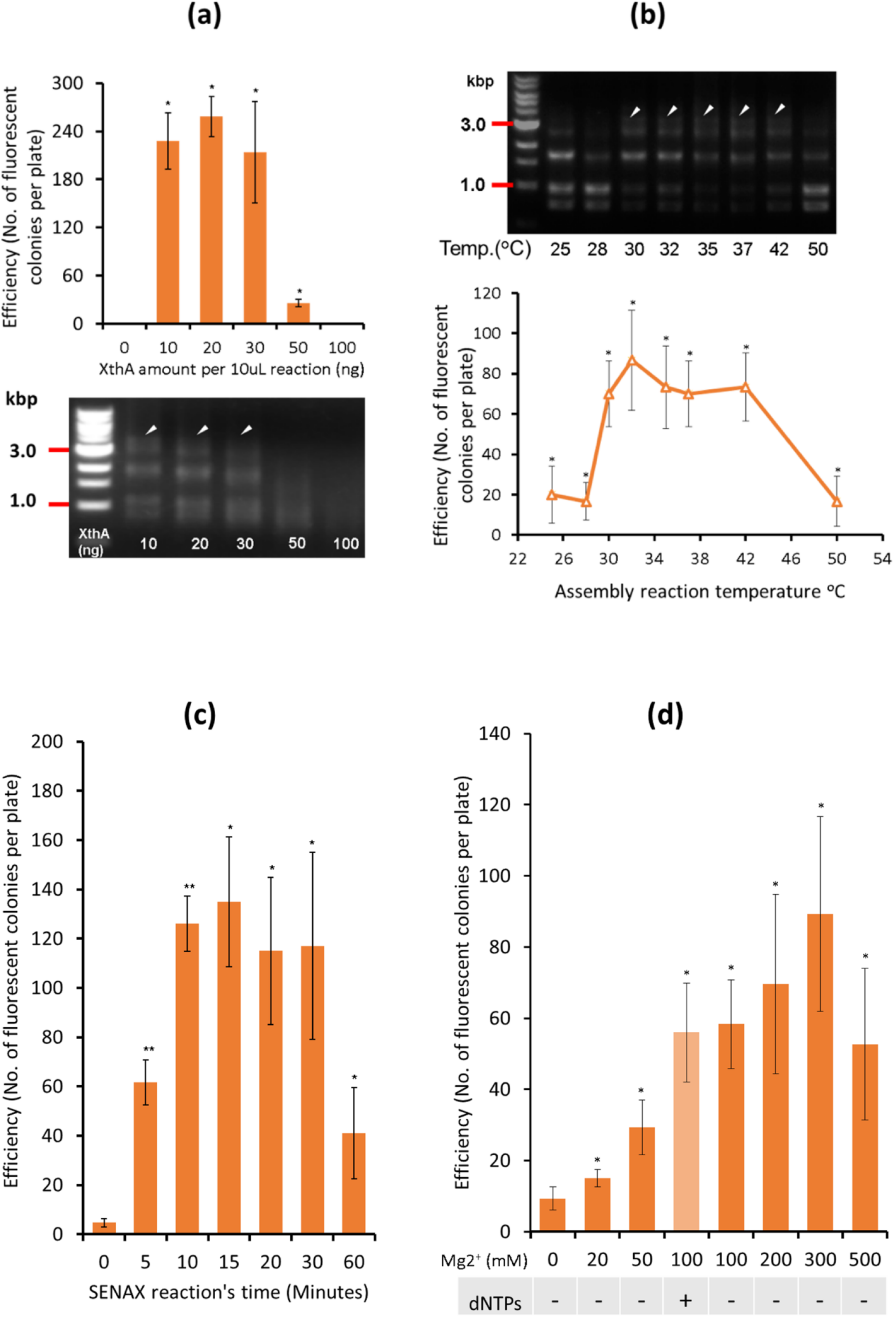
Optimization of SENAX. The configurations of a replication origin (15A), antibiotic resistance (AmpR), and a green fluorescence gene (Construct B) were used for assembly for all the tests. (**a**) Effect of Enzyme XthA amount on SENAX. Assembly mixes of the 3-fragment assembly reaction after 15 min with different XthA amounts were verified by agarose electrophoresis (below). Plot showing the efficiency of assembly along with the different amounts of XthA (above). The sample without XthA supplementation was used as the control. Arrows indicate the expected intermediate assembly product. The error bars represent the standard deviations (STDEVs) of three distinct replicates. *p < 0.05, **p < 0.01 by paired t test against the control (without XthA). (**b**) Effect of temperature on SENAX. Assembly mixes of 3-fragment SENAX assembly after 15 min incubation at different temperatures were verified by agarose gel electrophoresis (above). Plot showing the efficiency of assembly by SENAX at different temperatures after transformation (below). Arrows indicate the expected intermediate assembly product. The error bars represent the standard deviations (STDEVs) of three replicates. *p < 0.05, **p < 0.01 by paired t test against the control (without XthA). (**c**) Effect of incubation time on SENAX. 3-fragment assembly with different incubation times (0 min, 5 min, 10 min, 15 min, 30 min, 60 min). The error bars represent the standard deviations (STDEVs) of three replicates. *p < 0.05, **p < 0.01 by paired t test against the control (0 min incubation). (**d**) Effect of Mg2^+^ concentration on SENAX. 3-fragment assembly with different amounts of Mg2 + supplemented in the reaction. The error bars represent the standard deviations (STDEVs) of three distinct replicates. *p < 0.05, **p < 0.01 by paired t test against the control (without supplementation of Mg2 +).

We investigated the assembly efficiency using different amounts of XthA. We obtained similar efficiency when using 10–30 ng of XthA for a single reaction. In contrast, no fluorescent colonies were obtained when more than 50 ng of purified XthA was used in a single 10 µL reaction. The control sample with 0 ng of XthA showed no colonies, as expected. We further verified the assembly product by agarose gel electrophoresis. The faint bands that represented the final assembly product (approximately 3 kb) only appeared in the sample with 20 or 30 ng of XthA (Fig. 4a), which was consistent with the transformation-based results. Hence, 20 ng of XthA was found to be optimal for assembly, while 50 ng was the upper limit of the enzyme amount needed for a single 10 µL assembly reaction.

#### Effect of temperature on SENAX

We also investigated the effect of temperature on SENAX efficiency. The results show that SENAX produced colonies harbouring the assembled construct with almost similar efficiency when the reactions were performed in the temperature range of 30–42 °C (Fig. 4b). The number of fluorescent colonies significantly decreased when the incubation temperature was 50 °C or when the temperature was lower than 28 °C. The result suggests that the highest efficiency can be obtained at 32 °C, as the highest number of fluorescent colonies was obtained. At 35–37 °C, we obtained a similar number of fluorescent colonies compared with that at 32 °C. However, there were only a few colonies without fluorescence when the temperature was 32 °C, while the number of nonfluorescent colonies gradually increased when the temperature was reduced to 30 °C and lower. We performed sequencing analysis for some of the nonfluorescent colonies and found that these colonies had incorrect constructs with missing small DNA parts (data not shown). At 50 °C, few or no fluorescent colonies grew on the plate. For further analysis, an aliquot of assembly solution was verified by agarose electrophoresis (Fig. 4b). The DNA bands at approximately 1 kb represented the linear input DNA fragments. The DNA bands found from 1.5 to 2.0 kb probably represented the linear assembled product, in which only 2 DNA fragments were concatenated. Above those bands, we found bands at approximately 3.0 kb, representing the intermediate circular construct. As we only found these intermediates in the profile of samples at 30–42 °C, this is consistent with what we obtained from the screening of colonies on plates after transformation. The remaining linear input fragments after reaction in samples 30–42 °C were also much less than those of samples incubated at 25 °C, 28 °C, and 50 °C. We concluded that these temperatures inhibit enzyme activity and that 50 °C would likely deactivate XthA. In summary, the temperature for assembly using XthA was optimal between 32 °C and 37 °C.

#### Effect of incubation time on SENAX

To test the influence of reaction time on XthA assembly activity, we performed parallel assembly reactions joining the 3 DNA fragments for different incubation times. The results show that 10 to 30 min is the best incubation duration for cloning efficiency (Fig. 4c). Shorter than 10 min of incubation greatly decreased the efficiency, as approximately 2 times less activity was observed. The 60 min incubation time sharply decreased efficiency. The percentage of fluorescent colonies was reduced by more than 70% compared with the experiment using a 15 min incubation time. These results suggest that 10–30 min are suitable for DNA assembly by XthA.

#### Effect of Mg2 + concentration on SENAX

Structural analysis of XthA revealed that this enzyme has single divalent metal ion and nucleotide binding sites at the active site of the enzyme^8^. It was reported that Exo III catalyzed the stepwise removal of mononucleotides from the 3′-end in an Mg2 + -dependent manner^8,20^. Among the divalent cations, Mg2 + is the preferred ion for various enzymes dealing with DNA digestion, and we investigated the dependency of the activity of SENAX on this ion. To this end, we performed parallel reactions to assemble the 3 DNA fragments using different final MgCl_2_ concentrations, from 0 to 500 mM (Fig. 4d). The results show that with increasing Mg2 + concentration in the assembly reaction, the efficiency gradually increased until the concentration of 300 mM. The efficiency obtained by a 500 mM final concentration of Mg2 + decreased 40% from that of the 300 mM sample and was lower than those of the 100 mM and 200 mM samples. In addition, dNTP supplementation in the reaction with 100 mM Mg2 + did not affect the assembly efficiency. This result demonstrated that the presence of dNTPs has no effect on SENAX, which relies only on exonuclease activity. The Gibson method uses a Phusion DNA polymerase and requires dNTPs for this enzyme activity. The In-Fusion method uses a polymerase with exonuclease activity to accomplish the reaction^22,23^. Without any dNTPs added to the reaction, SENAX is clearly active without polymerase activity. The experiment also revealed that without Mg2 + supplementation, weak assembly activity was observed. This is probably due to the traces of divalent cations that were originally present in the DNA substrate.

#### Effect of size of the homology region

The typical length sequence needed for annealing in a PCR is 18 bp. Therefore, the length of the cloning primer, which should include the homology arm shorter than 20 bp, can be approximately 33–38 bp. This length is generally accepted as a fine balance between specificity and amplification efficiency. The long homology region (e.g., 30–40 bp homology as in the typical Gibson method) will require more cost and increase the chance of DNA mispriming and more likely result in an unexpected construct. Therefore, to reduce the possibility of mispriming and the presence of unexpected constructs due to the long homology arm, we designed the length of the homology region in our bioparts to be shorter at 18 bp. From most of the experiments performed, we demonstrated that 18 bp homology works well for SENAX. We also tested using 15 bp homology arms (e.g., for the Naringenin plasmid assembly and the overhang test) (Fig. S8b) and using 16 bp homology arms for short-fragment assembly. We found that the homology length can be as low as 15 bp without affecting cloning efficiency. Since the length of the homology arm will affect the annealing of the exonuclease-generated overhangs, the short homology is also suitable for the temperature used in SENAX (30–37 °C) rather than the 50 °C used in Gibson and In-Fusion. To determine the lower limit of the homology arm size using SENAX *in-vitro* DNA assembly, the DNA assembly of 3 fragments (Amp, 15A, GFP) with different overlapping lengths between the fragments (18 bp, 15 bp, 12 bp, 10 bp) was investigated (Fig. S10). The results show that the efficiency of the reaction decreased with the decrease in the size of the homology arm. However, the number of colonies obtained by the 12 bp homology arm was reduced by approximately 70% compared with the fluorescent colonies obtained with the 15 bp homology arm. The 10 bp homology arm produced only a few fluorescent colonies. Hence, 10 bp homology can be considered the lower limit for SENAX design. Nonetheless, using the 12 bp and 10 bp homology arm sizes still yielded fluorescent colonies, thereby showing that the SENAX method works even with smaller homology arm sizes. Overall, 15–18 bp can be considered the optimized length of the homology arm for SENAX assembly.

#### The effect of the blunt end, sticky end, 3′-prime overhang, and 5′-primer overhang inserts on SENAX

We tested the cloning of the blunt end and sticky end (3′-prime overhang and 5′-prime overhang inserts) using SENAX (Fig. S8). The inserts were amplified by PCR with specific primers that harbour either restriction site of XbaI with BamHI or XbaI with KpnI at the 2 terminals of the inserts. The amplicon was then treated with the corresponding restriction enzyme to release the 5′–5′ overhang fragment (XbaI-BamHI) and 5′–3′ overhang fragment (XbaI-KpnI) (Fig. S8a). The results showed that the efficiency of blunt-end insert cloning was the highest, followed by 5′–5′ overhang insert cloning, while there were no colonies in the sample with a 5′–3′ overhang (Fig. S8a). It can be assumed that the 3′-overhang fragment remained undigested upon completion of the incubation time. The same phenomenon was obtained when we performed a test of overhang’s influence on short-fragment assembly (Fig. S8b), in which we obtained 37, 33, and 0 colonies for samples of the blunt end, 5′-overhang and 3′-overhang short fragments, respectively. This is consistent with the literature reporting on the activity of exonuclease III, in which the enzyme was described to not actively work on single-stranded DNA, as the 3´-protruding termini (over 4 bp) are resistant to cleavage^24^.

## Discussion

XthA is known as a multi-functional enzyme, and its homologues are involved in the DNA repair system in various bacterial species. Nonetheless, XthA has been applied to several in vitro applications, including the analysis of protein-DNA complexes^25–28^. The controlled XthA digestion of DNA fragments can be used for sequence analysis of short DNA fragments. We realized that this “limited” exonuclease activity of XthA is unique and can be explored for other applications. In this study, we reported a new method to use XthA for in vitro DNA assembly, followed by *E.coli* transformation. Interestingly, using this enzyme is sufficient for the DNA assembly reaction not only for multiple DNA fragments but also for enabling the assembly of short fragments. While XthA has been studied in earlier studies in in vivo DNA assembly^13,15,16^, in this study, we have gone further in developing an in vitro DNA assembly method that uses only XthA to achieve the efficient assembly of multiple fragments, including short fragments. With SENAX, the homology ends are digested by XthA and annealed in vitro, while the resulting intermediates are being repaired (gap filling and covalently bonding) presumably inside the *E.coli* cells after transformation. A key feature of SENAX is that it requires short overlapping homology arms as short as 12–18 bp.

We demonstrated that SENAX can assemble up to 6 DNA fragments, and the length of the final construct can vary from 0.1 to 10 kb. This method has succeeded in producing a high success rate of correct colonies with design matched sequences, demonstrating the overall accuracy of the developed method. Importantly, SENAX allows short fragments from 200 bp down to 70 bp to be inserted into the template construct (from a few kb to 9.0 kb) in a single step. This overcomes the difficulty faced by the use of currently available homology-based assembly techniques for short fragment assembly. When we applied SENAX for promoter-RBS short fragment assembly, although the efficiency was relatively not as high as that of the medium-sized fragment assembly, correct colonies were obtained in the tested cases, while Gibson and In-Fusion gave almost no colonies. The difficulty of other homology-based assembly technologies in dealing with these fragments could be due to the short DNA and/or the nicked DNA being degraded much faster by T5 exonuclease (in Gibson mix)^29^. The T5 exonuclease could chew through an entire fragment shorter than 200 nucleotides before the annealing steps could occur^2^. A similar situation could be assumed for the exonuclease enzyme used in In-Fusion technology. Meanwhile, the nicked DNA substrate is known to be a weak substrate for XthA compared to other exonucleases^20^. This enzyme does not attack the single stranded DNA since the hydrolysis is specific for base-paired nucleotides in this enzyme^24^. In a practical report with duplex DNA, the enzyme XthA stops degradation when 35% to 45% of the nucleotides have been hydrolyzed and leaves a number of base-paired nucleotides undigested^30^. A recent study applied XthA to digest short DNA sequences without destroying the hairpin structure^31^. Nevertheless, XthA was reported to have several specific retardation sites, limiting the degradation of DNA during a certain time of incubation^25^. More interestingly, XthA is a distributive enzyme that attacks dsDNA nonprocessively at 37 °C, dissociating frequently from the DNA strand during digestion^32^. Therefore, in the short-fragment assembly using SENAX, it could be possible that during the stepwise cleavage by XthA, the ss-tailed DNA could anneal with the short complementary ss-overhang of the backbone during the disassociation of XthA, generating the intermediate nicked/gap DNA circular plasmid. Because of the gaps presented in the intermediate circular construct, this substrate appeared to be resistant to further digestion by XthA. It is likely that the intermediate product can be stable throughout the assembly course and can be transformed into competent cells to be repaired in vivo and further amplified.

**Figure 5:**
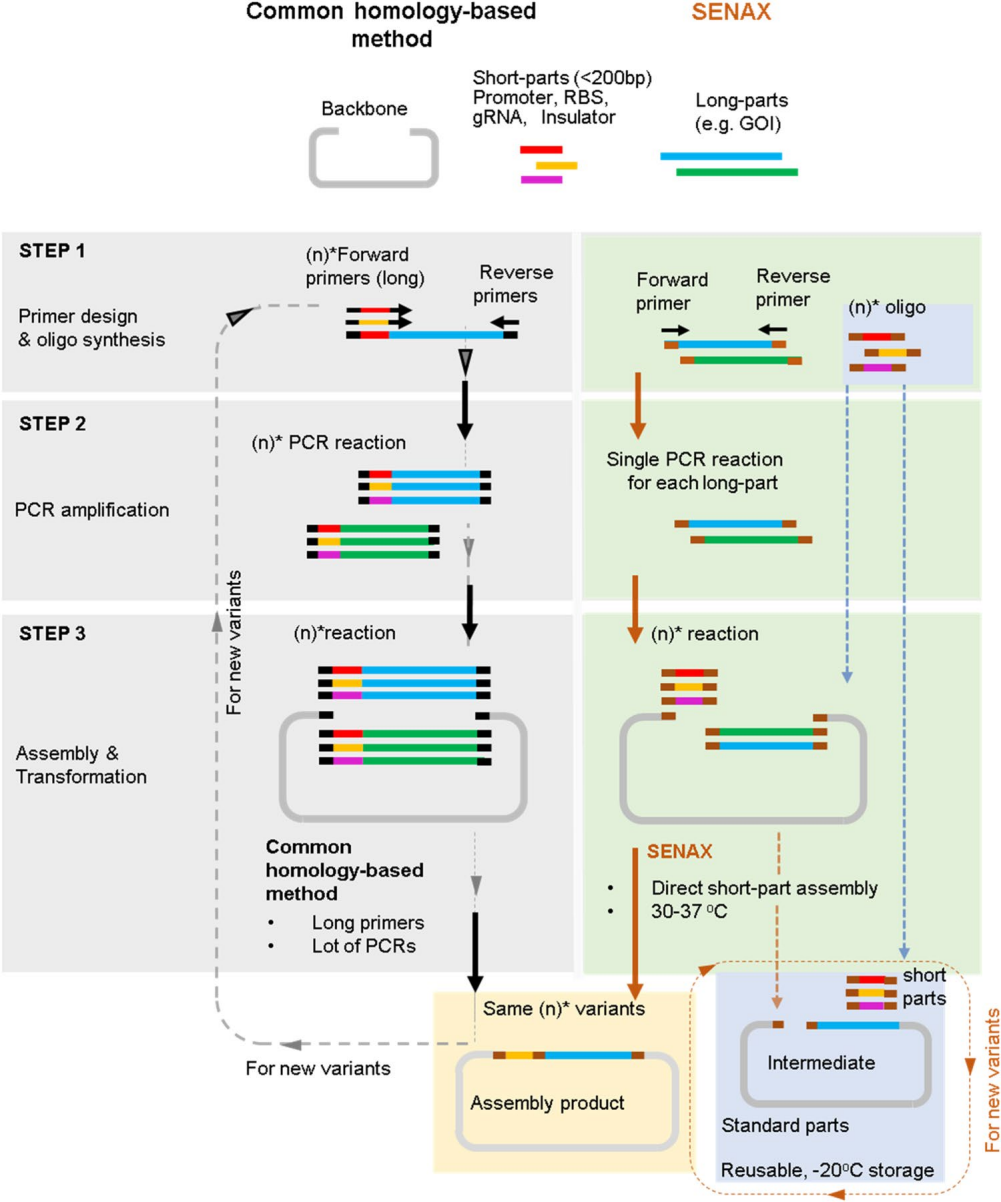
Illustration of SENAX versus commonly used homology-based methods to generate variants of short-fragment assembly constructs. n = the number of short parts to be incorporated. Because of the ability of SENAX to assemble short fragments directly into the backbone, the need for PCR long fragments to include the short fragment before assembly can be avoided. As a result, SENAX enables easier reuse of short fragments.

A series of repetitive steps are usually required using the current homology-based methods (e.g., Gibson or In-Fusion) to make the desired construct with the gene of interest accompanied by a specific promoter. As illustrated in Fig. 5, using the current methods, the primer that will include the whole short biopart (e.g., promoter) sequence upstream of the gene of interest will first need to be synthesized. After this, PCR will be performed to generate amplified products that harbour the desired short bioparts. However, this requires the use of long primers (usually 50–100 bp or more), resulting in a higher cost of DNA synthesis. In addition, because the Gibson assembly technique requires a longer overlapping region than other homology-based methods, even longer primers would be necessary. This could be considered a drawback of the Gibson assembly. If a fragment longer than 60 bp would be targeted, the length of the primer would not be suited for short oligo synthesis or would be difficult for PCR optimization. Alternatively, the intermediate template can be created by inserting the short target fragments directly into the original template using SENAX instead of resynthesizing the whole plasmid to achieve the complex construct. If only one plasmid is to be constructed, the cost between current homology methods and SENAX would be similar. However, in the event when the short fragments are commonly used and/or to be used in multiple constructs, using SENAX would enable the researcher to avoid the need to resynthesize the short fragment (considering that the amount of synthesized short fragment provided by the company is adequate for ~ 2000 reactions) but only need to synthesize the new short primer (~ 35 bp) to link the short fragment to the backbone. Therefore, the short fragments are considered “reusable”, which is where the cost is being saved compared with other typical methods. For example, using current methods, researchers would need to repeatedly synthesize new long primers (over 100 bp) to include the short fragment in advance. In other words, the more different constructs that the researcher needs to assemble using the short fragments, the more synthesis cost savings can be attained using the SENAX method.

Standardization of the assembly process is among the necessities for high-throughput DNA assembly. For sequence homology-based methods, one approach is to standardize the overlapping regions that are basically independent of the sequence of DNA parts. This will also allow the higher modularity of homology-based DNA assembly methods. In our study, the spacer (18 bp) was designed to facilitate the easy reuse of the bioparts. All constructs A, B, C, and D and their variants that differed from each other by only the promoter region were produced using this approach (Fig. 1b, Fig. S1, and Fig. S5a–d). Having 18 bp spacers for assembly also provides a means to positionally validate the assembled construct because these sequences can be used to design PCR primers. This PCR profiling approach provides a good marker to demonstrate the correct direction and order of bioparts in the final construct (Fig. S9). This also facilitates screening through colony PCR before sequencing, which is more convenient and cost-effective when applied in high-throughput assembly. It is worth noting that the number of spacers to guide assembly is not limited to 3 (as in our current 3-fragment vector design) but can be expanded for convenient use as long as more fragments are involved in the assembly.

As it is common practice to fine-tune gene expression by replacing promoters or RBSs, we developed a library of well-defined reusable DNA fragments of 88 bp to take advantage of SENAX’s capability to assemble short fragments. Each fragment is made up of commonly used constitutive promoters of varying strengths and RBSs. The specific set of promoters and RBSs in the proposed format can be reused in multiple constructs for various purposes (e.g., fine-tuning and combinatorial assembly). By using this approach, we were able to directly generate a library of construct variants that differ from each other by only the promoter region. This is advantageous, as the common homology-based technique would require starting over the whole plasmid construction in an ad hoc manner (Fig. 5). Overall, SENAX allows a standardized framework for reusing bioparts and improves the modularity for homology-based assembly (Fig. 5).

In this study, we designed our experiments such that we could compare the results within one experimental group when we varied one factor while keeping all the other factors fixed. However, it is difficult to keep all the factors fixed among the different experimental groups, including the competency of cells and the quality of DNA, due to the large number of samples required in the multiple experimental groups. It is known that cloning efficiency is critically affected by transformation efficiency, which is dependent on the competency of cells. Nonetheless, our experiments have enabled us to identify the optimal value of the factor studied. As a result, we elucidated several parameters required for SENAX. In particular, the SENAX workflow can be carried out flexibly with good efficiency for only 15 min from 32 to 37 °C, which is the temperature range used in common incubators widely available in laboratories. Notably, most of the commonly used homology-based methods will require a working temperature of 50 °C. Therefore, routine DNA assembly would be more convenient using SENAX for any laboratory, as water baths and hot blocks are no longer necessary. This temperature range would also be more compatible with a high-throughput automation system. In addition, SENAX comprises only a single exonuclease, while Gibson requires a polymerase, a T5 exonuclease, and a T4 ligase, and In-Fusion relies on a polymerase with exonuclease activity^1,6,33^. Polymerases have the possibility of generating sequence errors (mutation) and mismatches at the cloning junction in the final construct and will be more prone to introducing incorrect nucleotides at nonoptimal temperatures^34^. Having ligase increases the possibility of self-ligation of DNA parts that will introduce false-positive constructs that have incomplete bioparts^35,36^. Because no polymerase is involved in the assembly reaction, SENAX may avoid potential mutations at the junctions of the assembled plasmid. However, the possibility of mutations caused by polymerases during the PCR amplification of fragments is as likely as those caused by polymerases during assembly reactions. Having a single enzyme in the reaction of SENAX is also convenient for method optimization for automation and miniaturization, in comparison with methods based on multiple enzymes. Overall, this made the SENAX method easy to use, low energy consumption, and automation friendly.

## Methods

### Bacterial strains, culture conditions, and DNA materials

The strains and plasmids used in this study are listed in Table S1. Cells were cultured in LB medium (Axil Scientific Pte Ltd) containing the appropriate antibiotics at the designated temperature of 37 °C. In some experiments, the cultures were incubated at different temperatures for optimization purposes. The final concentrations of antibiotics ampicillin (Amp) (100 µg/mL), kanamycin (km) (50 μg/mL), chloramphenicol (Cm) (35 μg/mL), and spectinomycin (Spc) (50 μg/mL) were used for screening and maintaining plasmids in *E. coli*.

All the plasmids, DNA fragments, and primers used in this study were designed in silico using SnapGene (GSL Biotech) and Benchling (https://benchling.com). The primers used for the preparation of assembly fragments were designed to have a 12–18 bp homology region and are listed in Table S2. For the multifragment DNA assembly, 18-bp overlaps between fragments were designed. For the short fragment DNA assembly, a 16–18 bp overlapping region was designed. Genes and primers were obtained as gene fragments (gBlocks) or single-stranded oligos from Integrated DNA Technologies (IDT). GFP (green fluorescent protein), RFP (red fluorescent protein), and sfGFP (superfolding GFP) were used as reporters for gene expression characterization. Illustrations of the plasmids were prepared using SnapGene (GSL Biotech). The plasmids were constructed using the commercial enzyme mix Gibson (NEB), In-Fusion (Takara Bio), and the SENAX assembly method developed in this study. All constructed plasmids were chemically transformed into either *E. coli* Stellar (a brand derived from strain HST08) (Takara Bio), DH5alpha, or 10Beta (NEB). All protocols for transformations, PCR, and DNA manipulation used in this work were performed with reference to the manufacturer’s manual and were optimized when necessary.

### Standardized vector and preparation of DNA fragments for assembly test

Several plasmid variants were designed for the testing of the DNA assembly (Fig. S1). The plasmid format of the variants mainly comprises a configuration of DNA parts, including a replication origin (REP), an antibiotic resistance cassette (AbR), and a target gene of interest (GOI). Bioparts are linked by a random-sequence 18 bp spacer and can be produced by PCR amplification using either Q5 DNA polymerase (NEB) or KOD One PCR Master Mix (TOYOBO). Primers for amplification of the bioparts were designed based on junction sequences between spacers and bioparts. For the GOI of constructs A–D, we placed either a GFP or RFP reporter gene under the control of a constitutive promoter (e.g., J23101 from the Anderson promoter collection) and RBS0034, while REP and AbR were varied. These constructs were used for multifragment assembly and short-fragment assembly tests. A 2.8 kb reporter plasmid (construct B) was separated into 3, 4, 5, and 6 fragments by PCR for the multifragment assembly test. The DpnI-treated PCR-derived fragments were reassembled with SENAX. The sizes of the fragments were 750–1116–1029 bp (3 fragments), 750–719–415–1019 bp (4 fragments), 750–719–415–555–492 bp (5 fragments), and 750–719–415–249–324––492 bp (6 fragments). Constructs E (4 kb) and F (5 kb), which carried RFP and GFP, respectively, were used as templates for PCR preparation of 3 linear fragments for assembly to reproduce the original construct. Construct G (6.3 kb), which is a dCas9 expression plasmid, was used as a template for PCR preparation of fragments to produce the original plasmid and used for the short-fragment assembly test to produce its promoter variants. Construct H (10.4 kb), which is a naringenin-producing plasmid, was used as a template for PCR preparation of multifragments to produce the original plasmid and used for the short-fragment assembly test to produce its promoter variants. For the multifragment assembly test, this construct (H) was separated into 3, 4, 5, 6, and 7 fragments using PCR. The resulting amplicons from PCR preparation were treated with the restriction enzyme DpnI (NEB) to reduce the background of the circular DNA template, followed by purification in a gel (QIAGEN) or per aliquot by column (MACHEREY–NAGEL, Takara Bio USA).

### Screening of positive colonies and sequencing confirmation

The transformants were screened on antibiotic screening plates, and the extracted plasmids from several positive colonies were sent for sequencing (1st-BASE, Axil Scientific—Sanger sequencing service) to confirm the match to the designed constructs. The colonies with fluorescence reporters were also screened based on fluorescence that could be visualized with a trans-illuminator (GeneDireX, Inc.). The nonfluorescent colonies were screened by colony PCR.

### Production and purification of protein

The complete XthA gene sequence was directly cloned from a single colony of *E. coli* Stellar. The fully amplified 807 bp DNA fragment was purified using a gel extraction kit (Qiagen) and cloned into the linear blunt-end cloning vector pColdI, which was amplified by PCR, to yield plasmid pColdI::XthA (Fig. S2). The construct was introduced into *E. coli* Stellar, and the plasmid was isolated from the cells using a Miniprep kit (Qiagen). The inserted XthA and junctions were verified by nucleotide sequencing to confirm that the cloning was in frame. The correct plasmid was introduced into *E. coli* BL21 for protein expression. The cold-shock expression procedure using the pCold system allowed continuous translation of the histidine-tagged XthA gene product. The expressing culture was incubated at 37 °C until its absorbance at 600 nm reached 0.5. The culture was then placed on ice for 30 min. Isopropyl β-d-1-thiogalactopyranoside (IPTG) was added for induction at a final concentration of 1 mM for the next 16 h at 16 °C. The cells were then harvested and resuspended in PBS buffer. Cells were chemically disrupted by incubation with Tris–HCl-based lysis buffer with Triton X-100 (MERCK) for 30 min. The cell debris was removed by centrifugation (17000×g for 20 min) and filtration through a 0.22-µm membrane. The obtained cell extract was concentrated through 10-kDa cut-off filters (Millipore) by centrifugation at 5500×g until it reached an appropriate volume. An aliquot of concentrated cell extract was applied to a Ni–NTA spin column (Qiagen) for purification under the designated native conditions. In the last step, the buffer containing the purified protein fraction was changed to 50 mM Tris–HCl pH 7.5. The protein concentration was examined by NanoDrop One using Bradford reagent (BioRad).

### Sequencing analysis of expressed XthA protein

Approximately 1 µg was loaded onto SDS–PAGE (Tris–glycine 10% polyacrylamide gel). The single protein band appeared in the gel was then excised and dried using Vacuum Concentrator Plus (Eppendorf). The proteins were extracted from dried gel pieces and digested with trypsin, and the resulting peptide sequences were subjected to analysis (MALDI-TOF MS/MS—Proteomics International Laboratories Ltd, Australia).

### Testing the performance of enzyme mix by DNA assembly

For medium size DNA fragments, 18-bp homology arm length was designed; twenty (constructs A,B,C,D,E,F,G) or fifty nanograms (construct H) (ng) of each part was subjected to the mix for each assembly reaction. Except for short-fragment DNA assembly tests, an equal ratio of each part was used for each assembly reaction. For the short fragment, 500–1000 ng in 1 µL was supplied for each reaction. This depends on the original concentration of short-fragment stock synthesized by the company (IDT). Together with DNA parts, 20 ng in 1 µL of concentrated protein was mixed with 1 µL of buffer solution (100 mM MgCl_2_; 10 mM ATP; 10 mM DTT) on ice. Then, the reaction was filled to 10 µL with dH2O and incubated at the designated temperature. Unless otherwise indicated, the incubations were carried out at the designated 37 °C for 15 min.

To study the effect of the amount of XthA, temperature, reaction time and the effect of Mg2 + on the efficiency of the assembly, 3 fragment assemblies were performed using different amounts of XthA (0–100 ng) for each 10 µL reaction. The 3 fragments include one with a GFP placed downstream of the constitutive promoters J23101 and RBS0034 (GFP reporter), one with an antibiotic resistance gene (AmpR), and one with an origin of replication 15A (15A ori).

To identify the optimal temperature for the reaction, the reaction was performed at different temperatures (25 °C, 28 °C, 30 °C, 32 °C, 35 °C, 37 °C, 42 °C, and 50 °C), and the efficiency of the assembly was studied. A range of amounts of XthA, i.e., 5, 10, 20, 30, 50, and 100 ng, were also tested to optimize the method. The times evaluated for optimization were 0, 5, 10, 15, 30, and 60 min.

The resulting assembly mixtures (up to 10 µL) were verified by electrophoresis in a 1% agarose gel or chemically transformed into competent stellar cells (Takara), DH5 alpha (NEB), or 10beta (NEB). The transformed cells were preincubated at 37 °C for 1 h, plated on antibiotic screening plates, and incubated overnight. The resulting colonies were picked from overnight plates, and plasmid extraction (MiniPrep QIAGEN) was performed using 5 mL of fresh culture derived from a single colony.

### Assembly method for short fragment assembly

The short DNA fragments (single-stranded DNA oligos) were designed using SnapGene and purchased via IDT. The delivered dry oligos were suspended to a final concentration of 100 µM in water as the storage stock, and the two complementary oligos were mixed at a final concentration of 20 µM each. The obtained mixture was heated to 95 °C for 5 min and lowered to 4 °C at 0.1 °C/sec to allow annealing. The resulting duplex DNA solution was then kept at − 20 °C and was used for subsequent multiple different DNA assembly constructs. Approximately 500–1000 ng was used for each assembly reaction. Five short fragments of different lengths (200 bp, 150 bp, 100 bp, 88 bp, 70 bp) were designed (Table S2). All of the short fragments consist of spacer S1 at the 5′ terminus, a promoter, and an RBS. The capability and efficiency of assembling the short fragments into variants of the backbone template of different lengths (2.8 kb, 6.3 kb, and 9.0 kb) were investigated.

### Recommended SENAX assembly procedure

Linearized vectors/inserts are recommended to be generated by PCR, while chemically or enzymatically synthesized DNA fragments (e.g., gBlock) are also suitable for SENAX. To achieve successful assembly, the primers to generate a fragment via PCR must be designed in the way that its 5′-end contains bases (15–18 bp) of homology to the other fragment and its 3′-end must contain the sequence that can complementarily bind to the DNA template. Desalted oligonucleotide primers are suitable for PCR for SENAX. DpnI treatment of PCR products followed by gel purification is recommended to reduce the background derived from circular PCR template. In a final reaction mix of SENAX, two (2) ng/ul of XthA; 30 mM MgCl_2_; 1 mM ATP; 1 mM DTT and 1 to 10 ng/ul of each DNA fragment are suitable. With the assembly reaction for a DNA short-fragment, 50 to 100 ng/ul of short-fragment is suitable in the final reaction mix. The buffer that harboring chemicals and SENAX enzyme (XthA) should be thawed on ice before use. The reaction is carried out at 37 °C for 10 min and then terminates by placing on ice before transformation. The whole reaction can be used to transform into *E.coli* competent cell.

## Supplementary Information

Supplementary Information.
