# Supplementary material for: Single 3′-exonuclease-based multifragment DNA assembly method (SENAX)
Source: Sci Rep. 2022 Mar 7;12:4004. doi: 10.1038/s41598-022-07878-x (PMC8901738; doi:10.1038/s41598-022-07878-x)
Supplement: Supplementary file 1 — Supplementary Information. [file 41598_2022_7878_MOESM1_ESM.pdf]

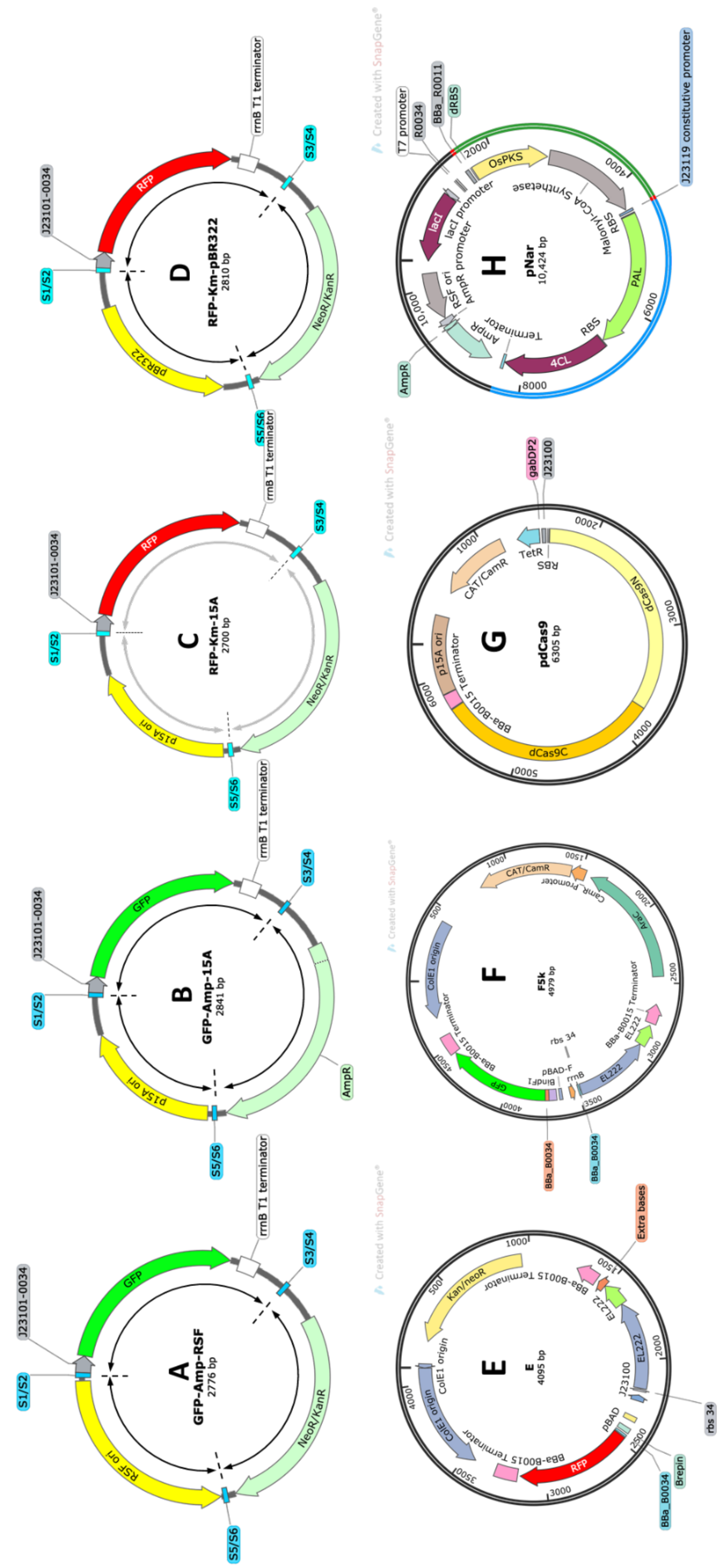

Figure S1

Figure S1. Genetic map of plasmids/configurations tested by assembly in this study.

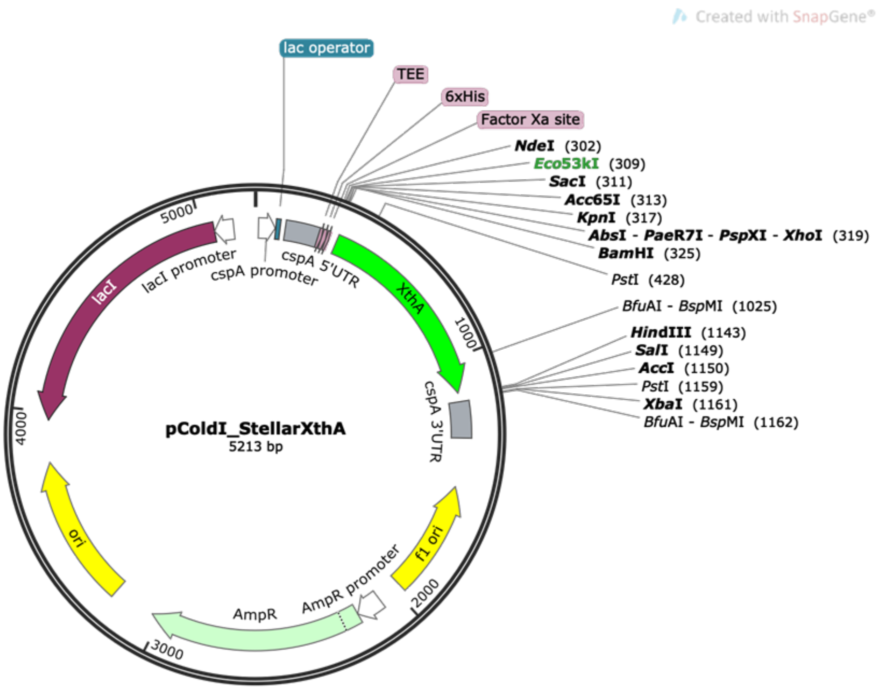


```
  1 MKFVSFNING LRARPHQLEA IVEKHQPDVI GLQETKVHDD MFPLEEVAKL
 51 GYNVFYHGQK GHYGVALLTK ETPIAVRRGF PGDDEEAQRR IIMAEIPSPL
101 GNVTVINGYF PQGESRDHPI KFPAKAQFYQ NLQNYLETEL KRENPVLIMG
151 DMNISPGDLD IGIGEENRKR WLRTGKCSFL PEEREWMERL MSWGLVDTFR
201 HANPQTADRF SWFDYRSKGF DDNRGLRIDL LLASQPLAEC CVETGIDYEI
251 RSMEKPSDHA PVWATFRR
```

Figure S2

Figure S2. Genetic map of plasmid pColdI harbouring XthA gene from *E.coli* Stellar (above). This plasmid was used for the expression of XthA enzyme.

To characterize the activity of XthA, we first constructed a plasmid, pColdXthA, to express Stellar *XthA* using *E. coli* BL21. The XthA product was purified using the crude cell extract. To verify the product, the obtained purified fraction was subjected to SDS-PAGE, and a single protein band corresponding to a molecular size of 35.0 kDa was obtained. The relative molecular size of this protein band was consistent with the deduced amino acid sequence of XthA gene with 6His-tag, TEE (translation enhancing element), and the Factor Xa cleavage site sequences that are originally from pColdI vector. Deduced amino acid sequence of *XthA* product with sequences confirmed by MALDI/TOF MS (below).

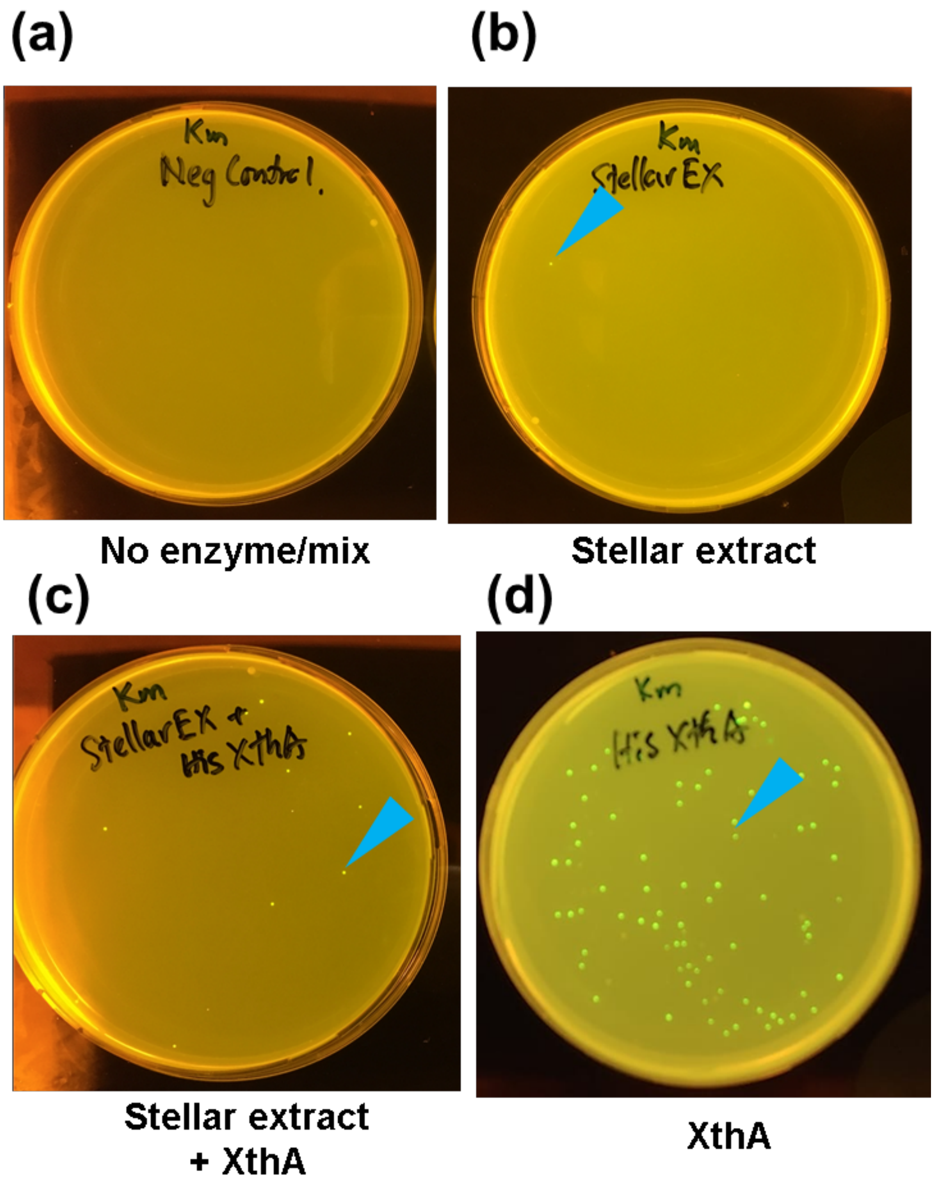

Figure S3

Figure S3. Images of plates after transformation with in-vitro 3 DNA fragments assembly by Stellar cell extract and/or XthA. Blue arrow indicated for an example of GFP colony.

We tested the efficiency of the XthA mix in assembling 3 typical medium-size fragments - a RSF origin of replication, a Kanamycin resistance, and a GFP reporter gene. We observed that the concentrated cell extract from Stellar had an innate activity to assemble DNA parts as a number of green fluorescent colonies grew on the screening plate (Fig. S3). This concurs with previously reported studies reporting SLiCE. Interestingly, when only the purified XthA was used for assembly, a significantly higher number of fluorescent colonies was obtained (Fig. S3). This result indicates that the single XthA was sufficient for DNA assembly. However, the efficiency was lower when we used concentrated Stellar cell extract with XthA supplemented. These results suggest that the cell extract probably contained some competitors to XthA, such as other dominant exonucleases in *E. coli* (RecBCD) that could inhibit the activity of XthA. For the sample which used only XthA, the number of fluorescent colonies was about 95% of the colonies grew on the screening plate. To confirm the sequences, three colonies among these fluorescent colonies were examined using sequencing. All the colonies sent for sequencing

have the correct sequences, suggesting that XthA can achieve high accuracy in DNA assembly. The samples with the same amount of DNA fragments but no enzyme XthA added were used as control. The controls had no colony on the screening plate, suggesting that *in-vivo* assembly is not effective.

(a)

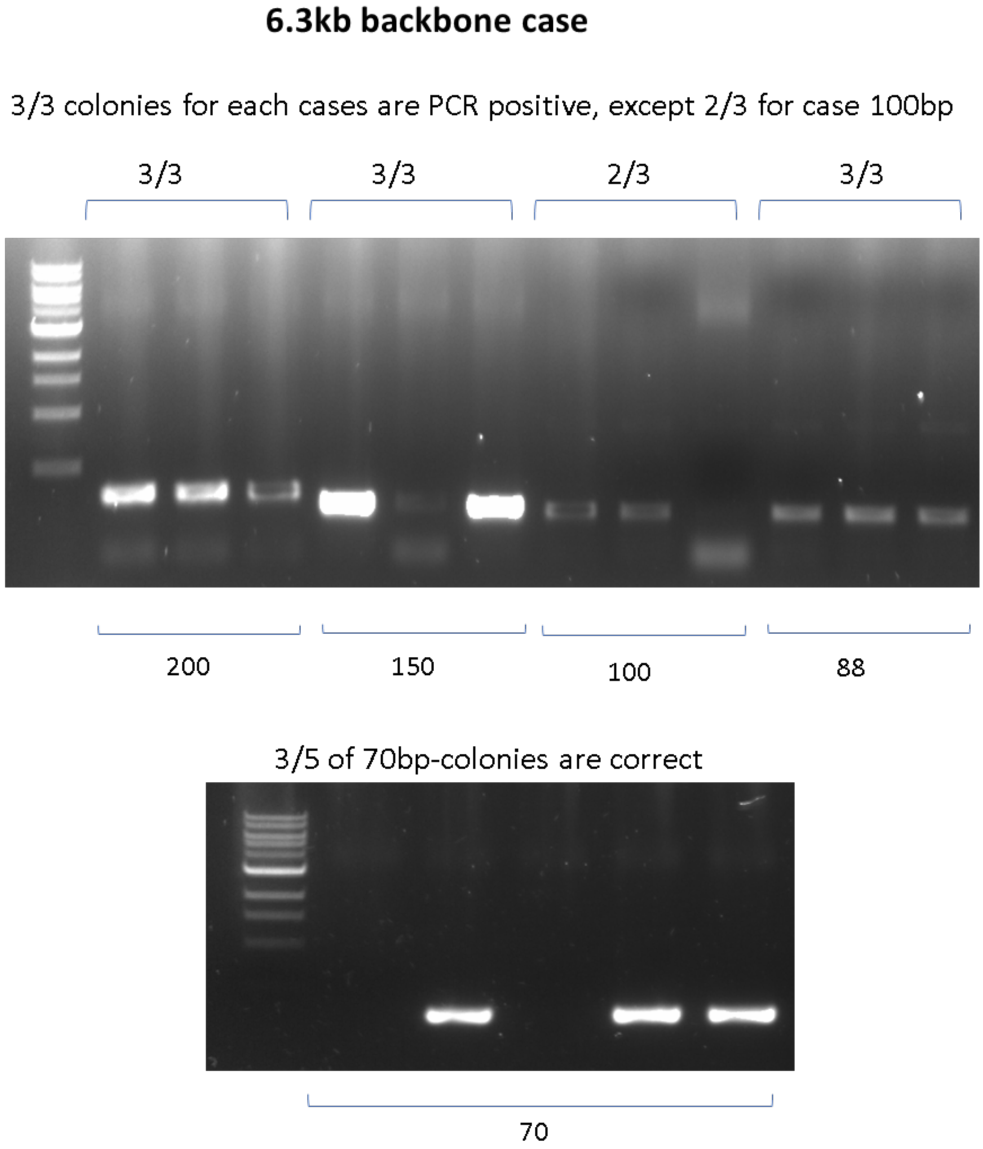

Figure S4

Figure S4. Evaluation of the accuracy of short-fragment assembly based on colony-PCR. (a) Experiment with 6.3kb backbone; (b) Experiment with 9.0kb backbone.

(b)

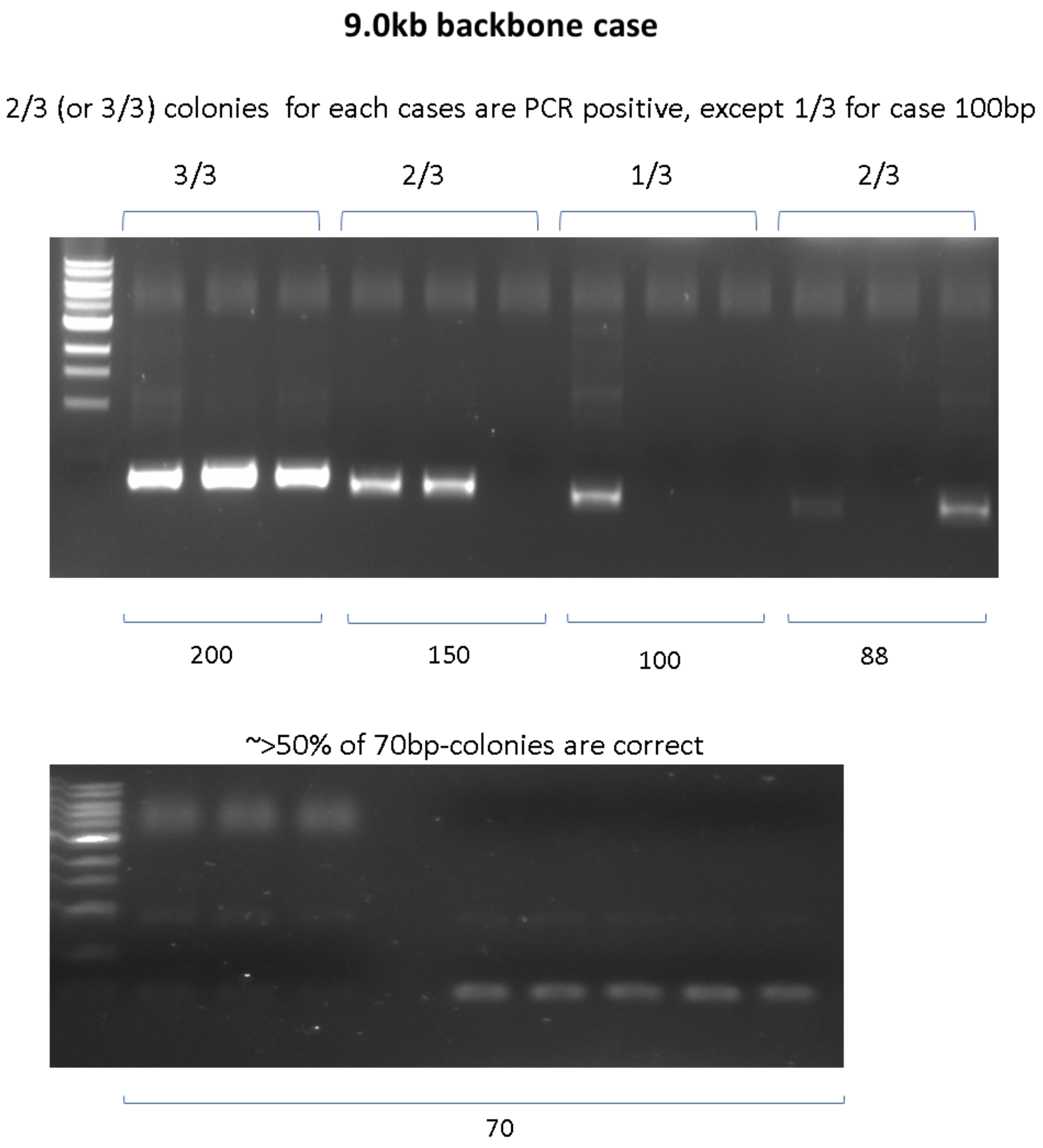

Figure S4

Figure S4. Evaluation of the accuracy of short-fragment assembly based on colony-PCR. (a) Experiment with 6.3kb backbone; (b) Experiment with 9.0kb backbone.

(a)

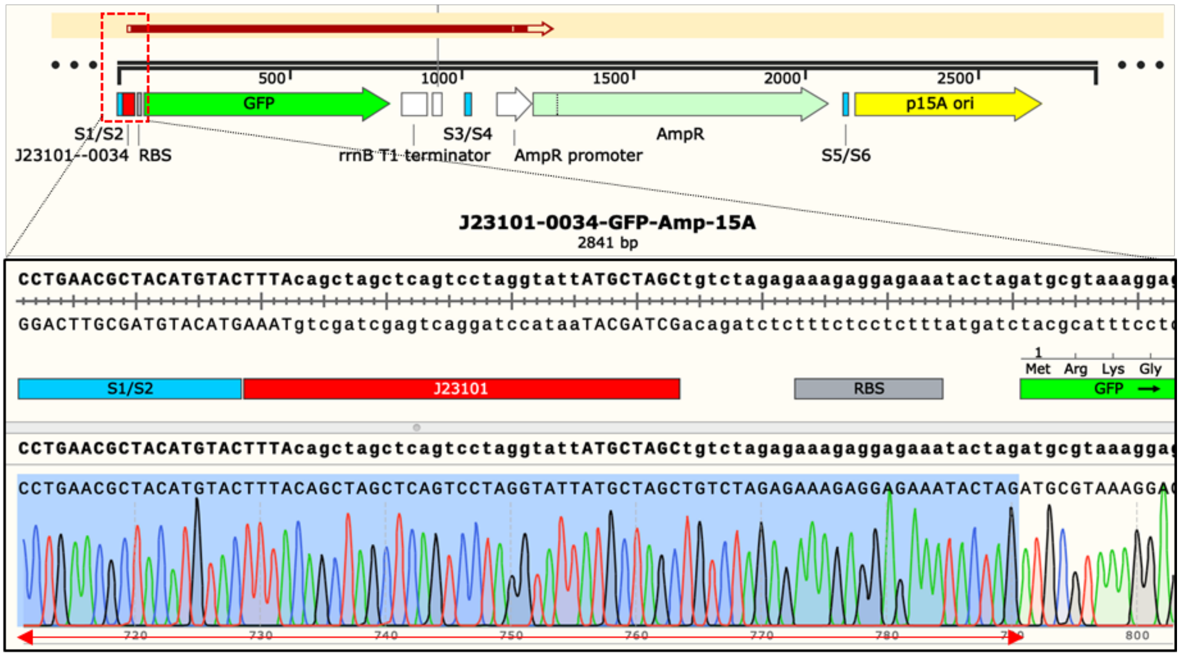

500
1000
1500
2000
2500
GFP
p15A ori
S1/S2
J23101--0034
RBS
rrnB T1 terminator
S3/S4
AmpR promoter
AmpR
S5/S6
J23101-0034-GFP-Amp-15A
2841 bp
CCTGAACGCTACATGTACTTTAcagctagctcagtcctaggtattATGCTAGCtgtctagagaaagaggagaaatactagatgcgtaaagga
GGACTTGCGATGTACATGAAATgtcgatcgagtcaggatccataaTACGATCGacagatctctttctcctctttatgatctacgcatttcct
Met Arg Lys Gly
S1/S2
J23101
RBS
GFP
CCTGAACGCTACATGTACTTTACAGCTAGCTCAGTCCTAGGTATTATGCTAGCTGTCTAGAGAAAGAGGAGAAATACTAGATGCGTAAAGGA
720
730
740
750
760
770
780
800

88bp

(b)

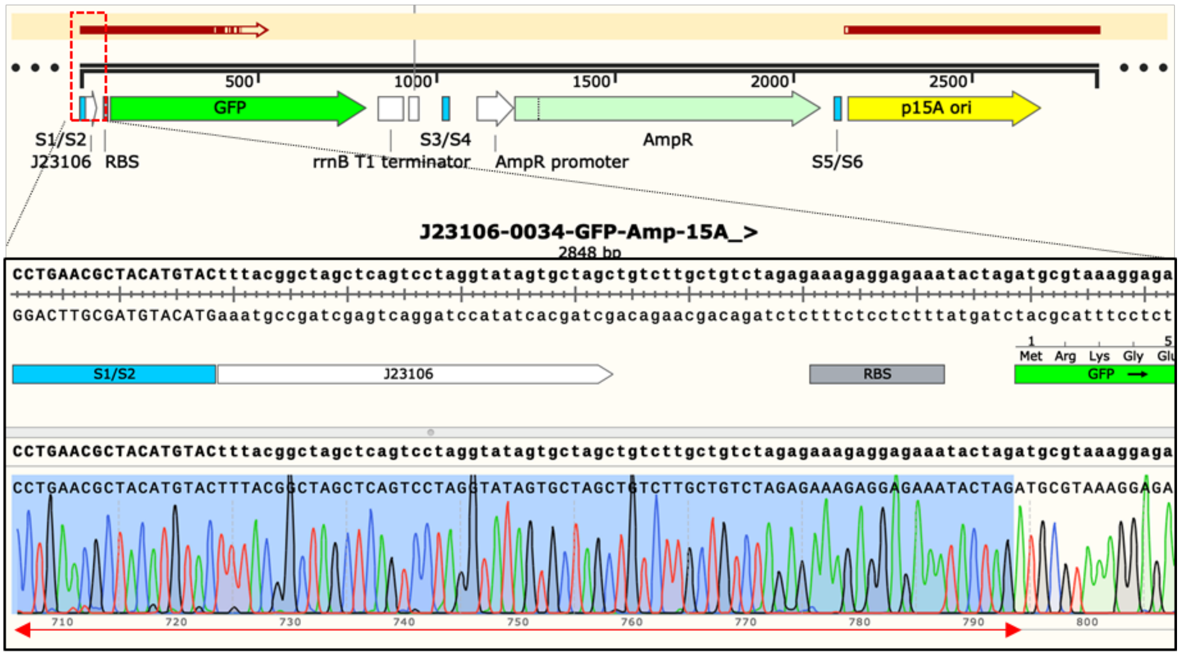

500
1000
1500
2000
2500
GFP
p15A ori
S1/S2
J23106
RBS
rrnB T1 terminator
S3/S4
AmpR promoter
AmpR
S5/S6
J23106-0034-GFP-Amp-15A_>
2848 bp
CCTGAACGCTACATGTACtttacggctagctcagtcctaggtatagtgctagctgtcttgctgtctagagaaagaggagaaatactagatgcgtaaaggaga
GGACTTGCGATGTACATGaaatgccgatcgagtcaggatccatatcacgatcgacagaacgacagatctctttctcctctttatgatctacgcatttcctct
Met Arg Lys Gly
S1/S2
J23106
RBS
GFP
CCTGAACGCTACATGTACTTTACGGCTAGCTCAGTCCTAGGTATAGTGCTAGCTGTCTTGCTGTCTAGAGAAAGAGGAGAAATACTAGATGCGTAAAGGAGA
710
720
730
740
750
760
770
780
790
800

88bp

(c)

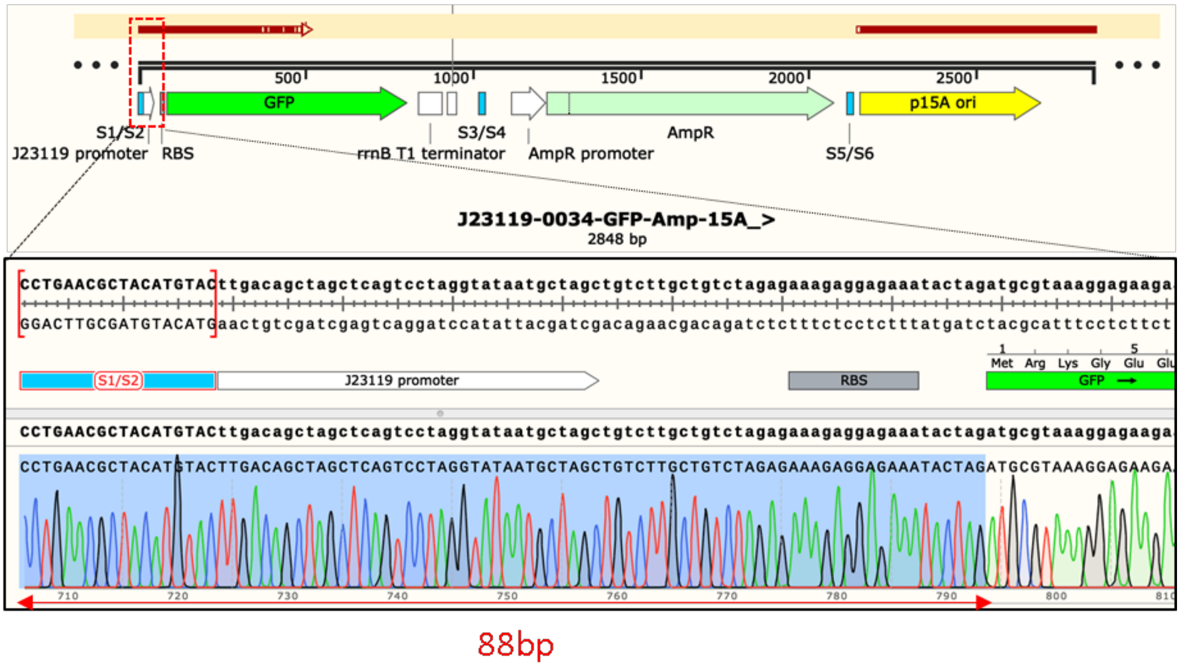

GFP
S1/S2
J23119 promoter
RBS
rrnB T1 terminator
S3/S4
AmpR
AmpR promoter
S5/S6
p15A ori
J23119-0034-GFP-Amp-15A_>
2848 bp
J23119 promoter
RBS
Met Arg Lys Gly Glu
88bp

(d)

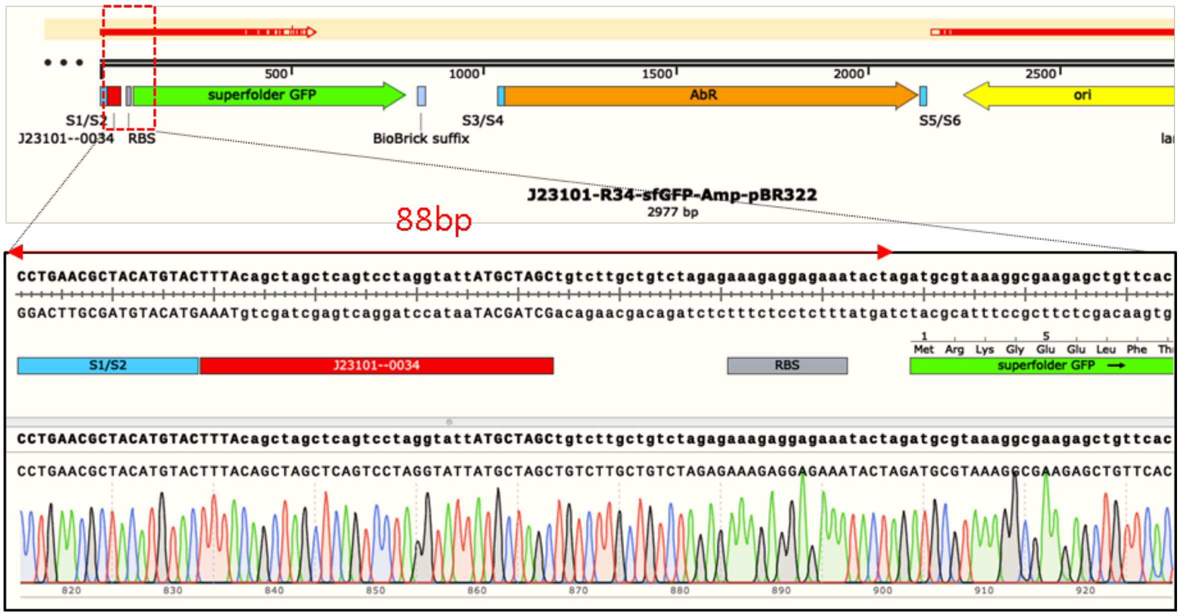

superfolder GFP
AbR
ori
S1/S2
J23101--0034
RBS
BioBrick suffix
S3/S4
S5/S6
J23101-R34-sfGFP-Amp-pBR322
2977 bp
88bp
Met Arg Lys Gly Glu Glu Leu Phe

(e)

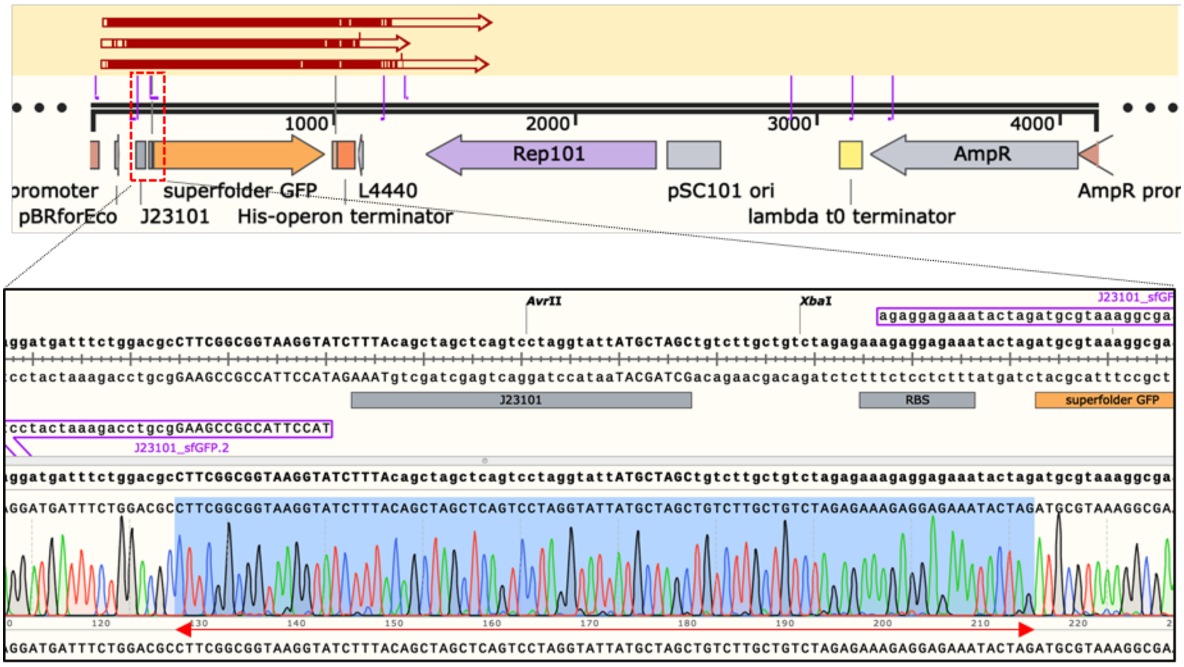

1000
2000
3000
4000
promoter
pBRforEco
J23101
superfolder GFP
His-operon terminator
L4440
Rep101
pSC101 ori
lambda t0 terminator
AmpR
AmpR pror
AvrII
XbaI
J23101_sfGF
J23101
RBS
superfolder GFP
J23101_sfGFP.2

88bp

(f)

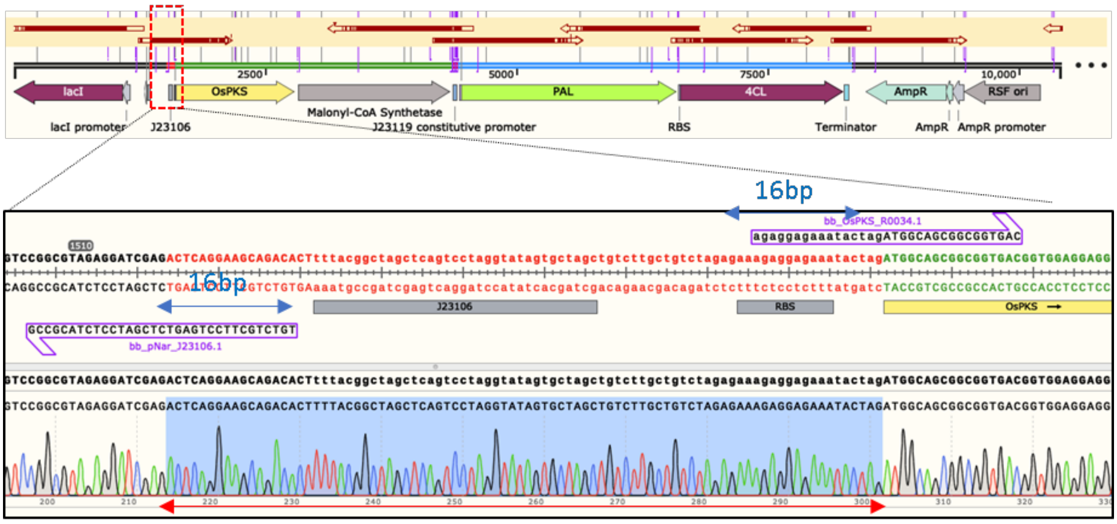

2500
5000
7500
10,000
lacI
OsPKS
PAL
4CL
AmpR
RSF ori
lacI promoter
J23106
Malonyl-CoA Synthetase
J23119 constitutive promoter
RBS
Terminator
AmpR
AmpR promoter
16bp
bb_OsPKS_R0034.1
16bp
J23106
RBS
OsPKS
bb_pNar_J23106.1

88bp

(g)

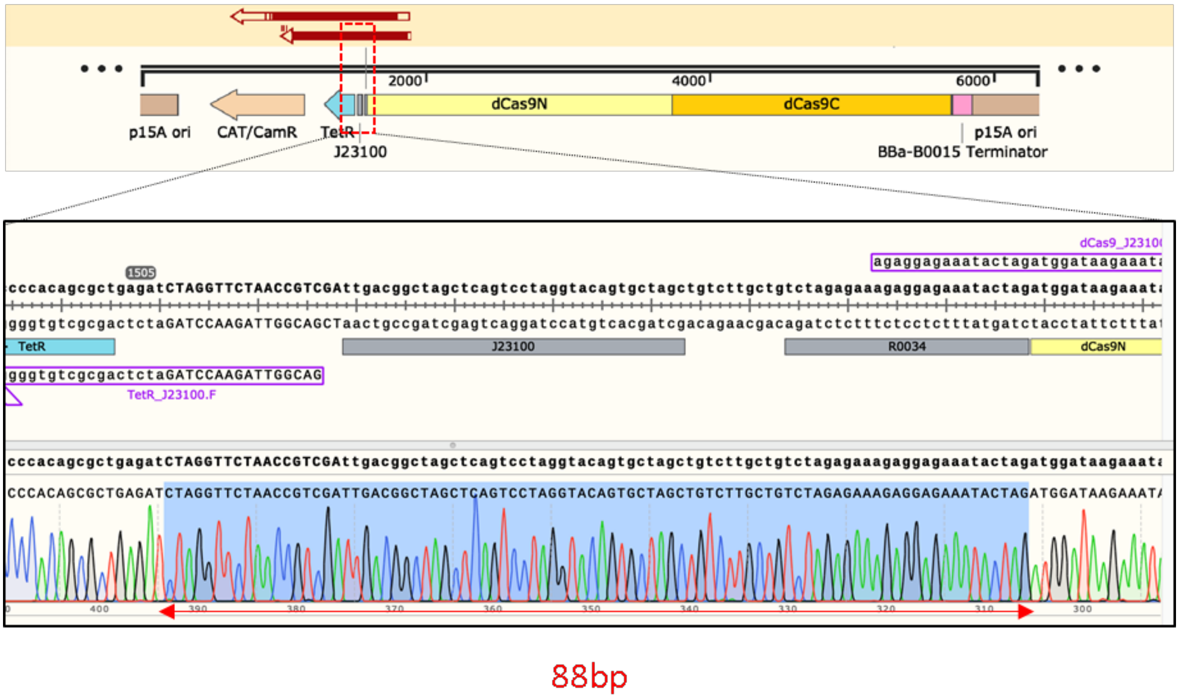

(h)

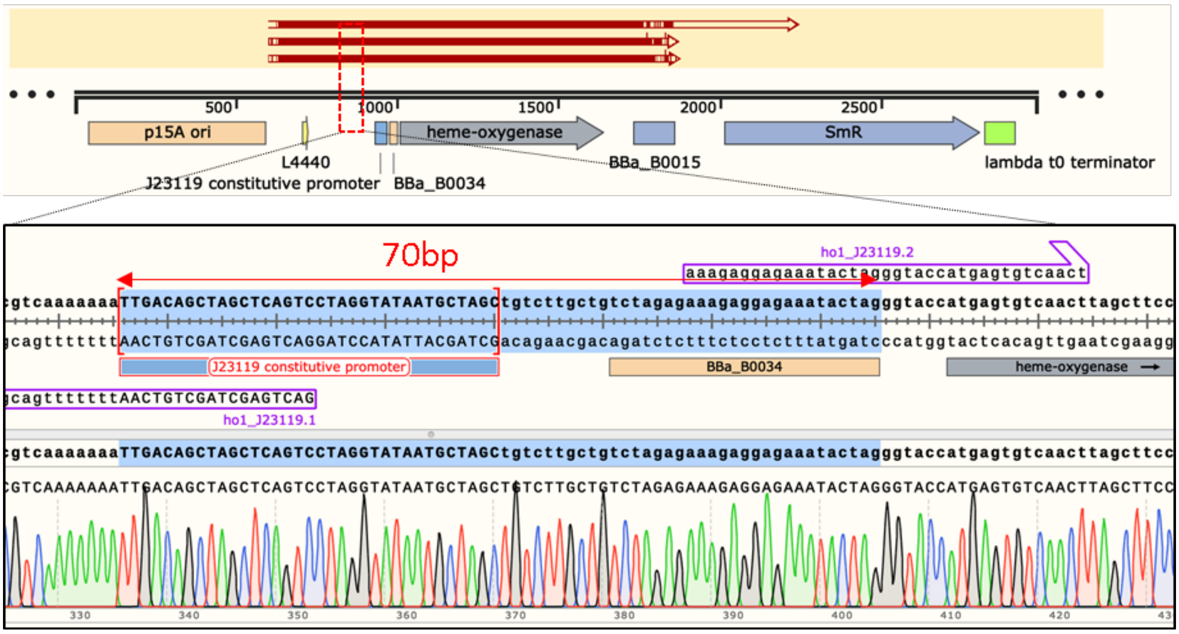

Figure S5

Figure S5. Short fragment exchangeability by SENAX. Detail DNA sequencing chromatograms of joint region and inserted part from resulted plasmids.
(a)(b)(c) Variants of GFP reporter with different promoters were generated by SENAX (J23101-0034-GFP-Amp-15A, J23106-0034-GFP-Amp-15A, J23119-0034-GFP-Amp-15A)

(d)(e) A set of a promoter J23101 and an RBS0034 (88bp) was placed upstream of sfGFP in a reporter plasmid (2.8kb) and (4.2kb)
(f) The original promoter – RBS region of Naringenin gene cluster was replaced by a new set of J23106-0034 (88bp)
(g) The original promoter – RBS region of dCas9 expression plasmid was replaced by a new set of J23100-00334 (88bp)
(h) The original promoter – RBS region of a heme-oxygenase producing plasmid was replaced by 70bp-fragment (a set of J23119 promoter with 0034 RBS). The 18bp homology arm was designed for DNA preparation.

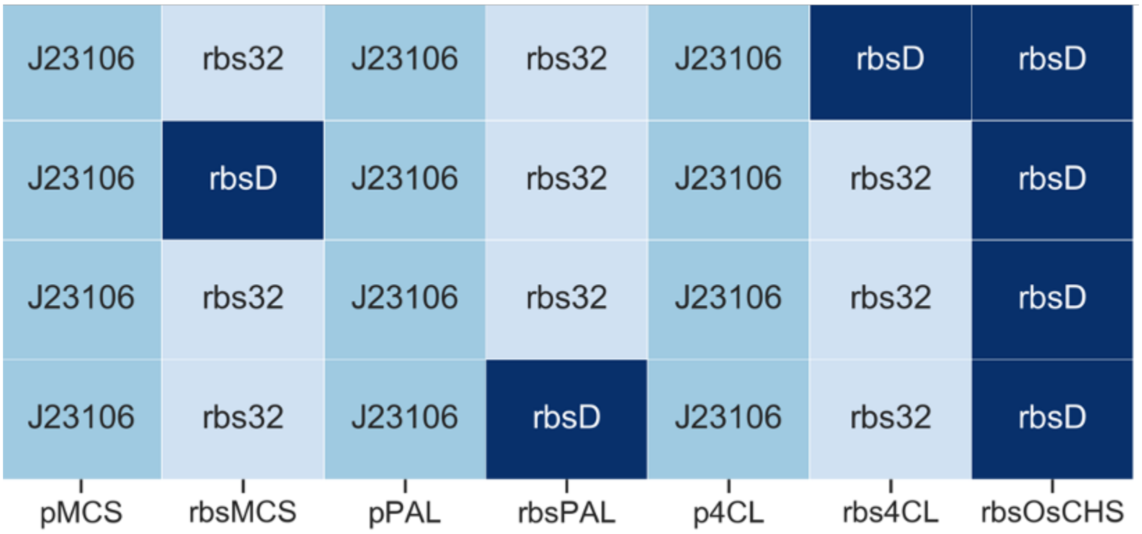

Figure S6

Figure S6. Illustration of combinatorial variants of Naringenin producing plasmid obtained by SENAX. MCS, PAL, 4CL, OsCHS are the GOIs

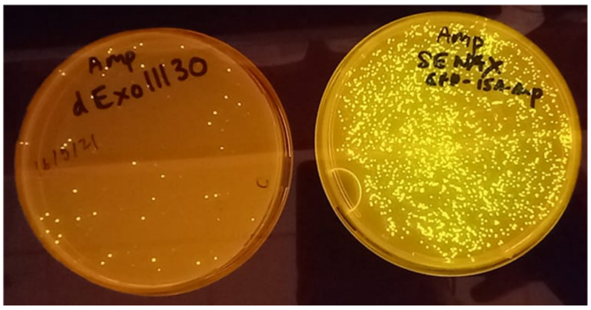

Figure S7: 3-fragments assembly using commercial XthA (NEB-M0206L). The configuration of a replication origin (15A), an antibiotic resistance (AmpR) and a green fluorescence gene (GFP) was used for the test. 100 ng of each fragment was used for the test. The left plate and right plate are samples with fluorescent colonies using commercial XthA (30 times dilution) and SENAX, respectively.

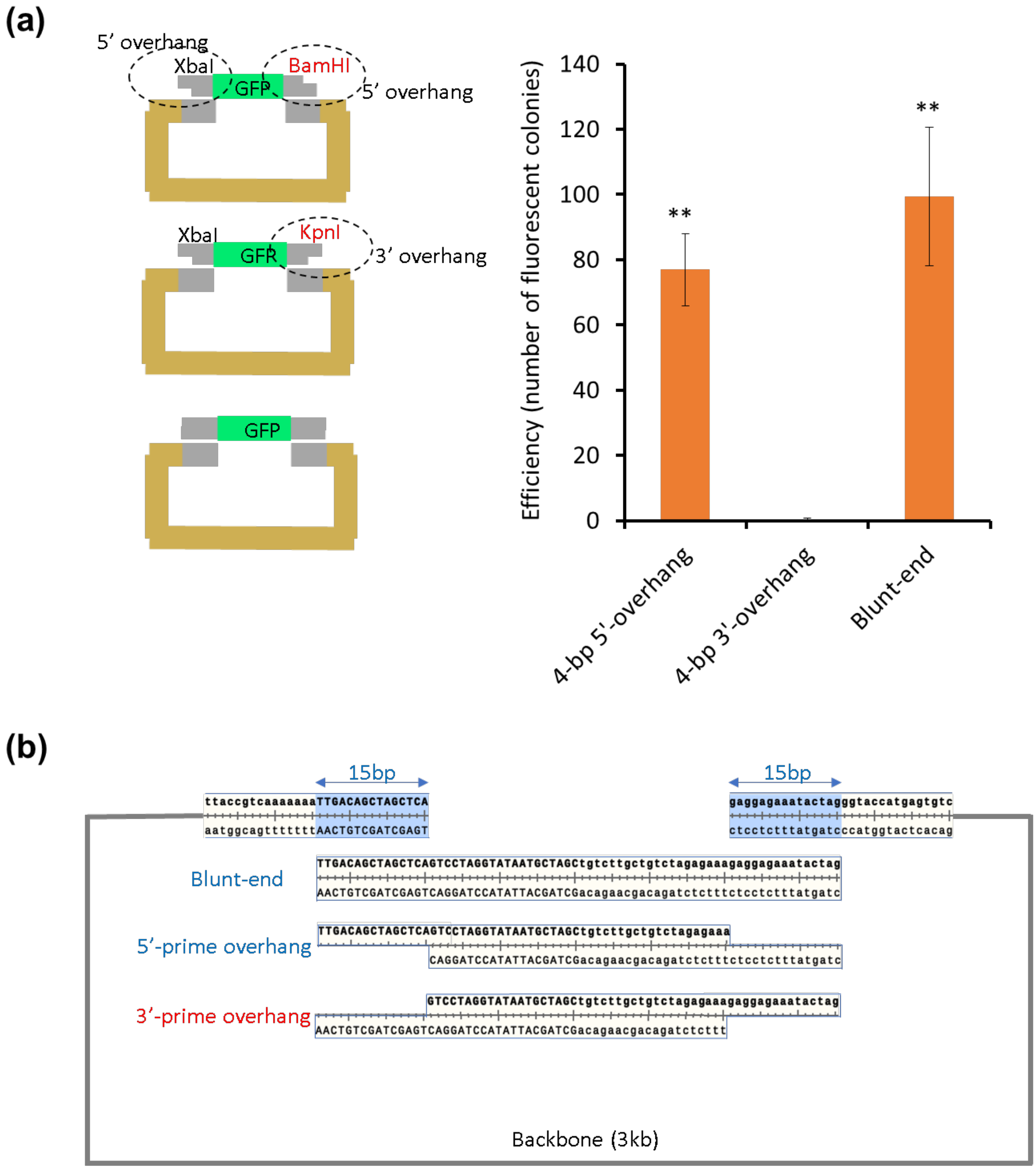

Figure S7

Figure S8. Overhangs fragment assembly tests. (a) Design for test of overhang fragment (mid-size) assembly. The inserts were amplified by PCR with specific primers that harbour either restriction sites of XbaI with BamHI or XbaI with KpnI, respectively at the 2 terminals of inserts. The amplicon was then treated with the corresponding restriction enzyme released 5’-5’overhang-fragment (XbaI-BamHI) and 5’-3’overhang-fragment (XbaI-KpnI). Evaluation of efficiency based on number of fluorescent colonies per plate. The error bars represent the standard deviations (STDEV) of three parallel replicates. *p<0.05, **p<0.01 by paired t-test against the controls. (b) Design for test for overhang short-fragment assembly.

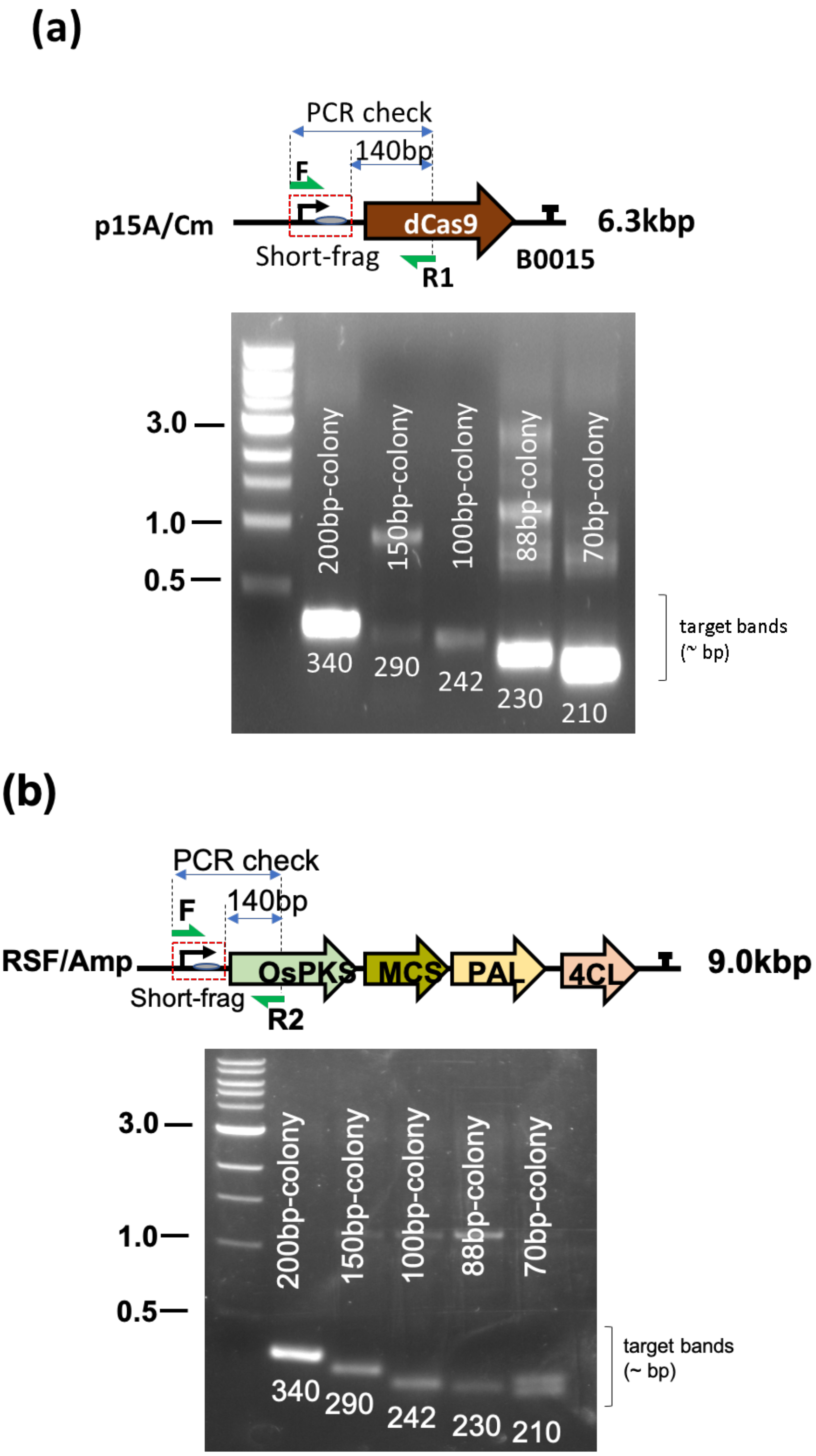

Figure S8

Figure S9. Colony-PCR for confirmation of short-fragment SENAX-assembly constructs with different short fragment inserted. (a) Experiment with 6.3kb backbone; (b) Experiment with 9.0kb backbone.

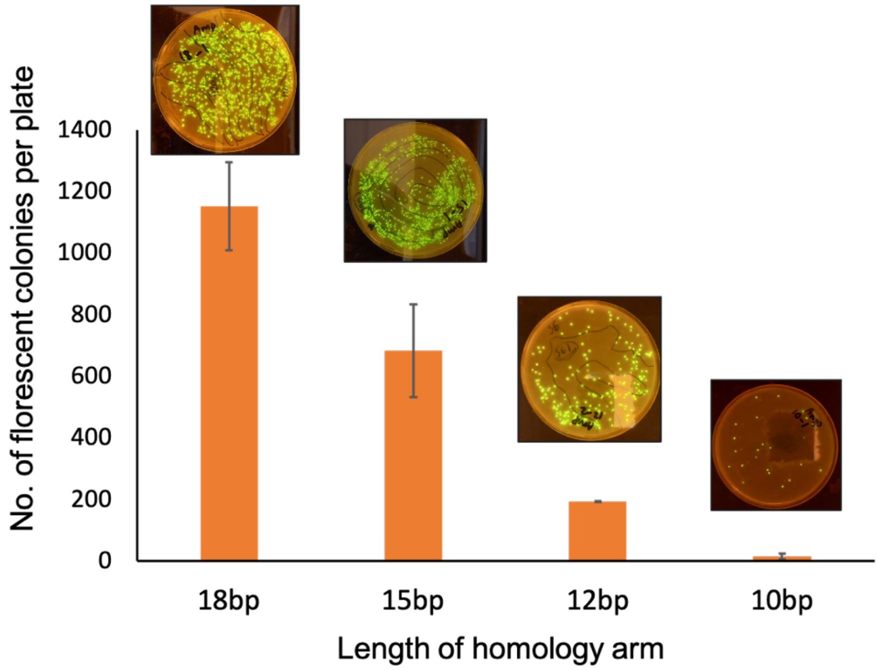

Figure S10: Effect of homology arm on SENAX invitro DNA assembly. 3-fragment assembly with different homology arm length (18bp-15bp-12bp-10bp). The configuration of a replication origin (15A), an antibiotic resistance (AmpR) and a green fluorescence gene (GFP) was used for the test. 100 ng of each fragment was used for the test. The error bars represent the standard deviations (STDEV) of two replicates. The images on top of each column are the representative images of the agar plate with fluorescent colonies obtained from the corresponding test conditions.

Table S1. Strains and plasmids used in this study

Plasmids	Strain	Description
pCold-SXthA	*E.coli BL21*	Expression plasmid for XthA
pbv-J23119-ho1	*E.coli* Stellar	Heme-oxygenase producing plasmid
pbv-J23107-ho1	*E.coli* Stellar	Heme-oxygenase producing plasmid
pNar4	*E.coli* Stellar	Naringenin producing plasmid
pdCas9	*E.coli* Stellar	dCas9 expression plasmid
pGFP-Amp-RSF	*E.coli* Stellar	Constitutive GFP expression plasmid
pGFP-Amp-15A	*E.coli* Stellar	Constitutive GFP expression plasmid
pRFP-Km-pBR322	*E.coli* Stellar	Constitutive RFP expression plasmid
pRFP-Km-15A	*E.coli* Stellar	Constitutive RFP expression plasmid
pGFP-Km-15A-J23101	*E.coli* Stellar	Constitutive GFP expression plasmid
pGFP-Km-15A-J23106	*E.coli* Stellar	Constitutive GFP expression plasmid
pGFP-Km-15A-J23119	*E.coli* Stellar	Constitutive GFP expression plasmid
	E.coli Stellar	Competent cell (Takara Bio- #ST0213))
	E.coli DH5Alpha	Competent cell (NEB-#C2987I)
	E.coli 10Beta	Competent cell (NEB-#C3019H)

Table S2. The synthetic oligos used in this study

Oligos	Oligo sequences (5'-3')	Purpose
S1	CCTGAACGCTACATGTAC	Spacer
S2	GTACATGTAGCGTTCAGG	Spacer
S3	CACTAGGCCAACAATAGG	Spacer
S4	CCTATTGTTGGCCTAGTG	Spacer
S5	ACGTAGCCTTGTAGTTAG	Spacer
S6	CTAACTACAAGGCTACGT	Spacer
S1_GFP	CCTGAACGCTACATGTACTTTACAGCTAGCTCAGTC	To amplify parts (Standard vector)
S3_Amp	CACTAGGCCAACAATAGGTACGCCTATTTTTATAGG	To amplify parts (Standard vector)
S3_Km	CACTAGGCCAACAATAGGGGAATTGCCAGCTGGGGC	To amplify parts (Standard vector)
S3_Cm	CACTAGGCCAACAATAGGGAAGCCCTGCAAAGTAAA	To amplify parts (Standard vector)
S3_Spc	CACTAGGCCAACAATAGGTGAGGATCGTTTCGTATG	To amplify parts (Standard vector)
S5_RSF	ACGTAGCCTTGTAGTTAGCAGCGCTCTTCCGCTTCC	To amplify parts (Standard vector)
S5_f1	ACGTAGCCTTGTAGTTAGGATTGTACTGAGAGTGCA	To amplify parts (Standard vector)
S5_pUC	ACGTAGCCTTGTAGTTAGTAATACGGTTATCCACAG	To amplify parts (Standard vector)
S5_pBR322	ACGTAGCCTTGTAGTTAGGTTATCCACAGAATCAGG	To amplify parts (Standard vector)
S5_15A	ACGTAGCCTTGTAGTTAGATTAATAAGATGATCTTC	To amplify parts (Standard vector)
S5_pSC101	ACGTAGCCTTGTAGTTAGTTGAAAACAACTAATTCA	To amplify parts (Standard vector)
S4_GFP	CCTATTGTTGGCCTAGTGGATAACCGTATTACCGCC	To amplify parts (Standard vector)
S4_RFP	CCTATTGTTGGCCTAGTGTGATTCTGTGGATAACCG	To amplify parts (Standard vector)
S4_sfGFP	CCTATTGTTGGCCTAGTGTCACCATGAACAGATCGA	To amplify parts (Standard vector)
S6_Amp	CTAACTACAAGGCTACGTCAATCTAAAGTATATATG	To amplify parts (Standard vector)
S6_Km	CTAACTACAAGGCTACGTAAGCGAGCTCTCGAACCC	To amplify parts (Standard vector)
S6_Cm	CTAACTACAAGGCTACGTCCAAGCGAGCTCGATATC	To amplify parts (Standard vector)
S6_Spc	CTAACTACAAGGCTACGTGATTCTCACCAATAAAAA	To amplify parts (Standard vector)
S2_RSF	GTACATGTAGCGTTCAGGGAAATCTAGAGTAACGGA	To amplify parts (Standard vector)
S2_f1	GTACATGTAGCGTTCAGGTTACGCATCTGTGCGGTA	To amplify parts (Standard vector)

S2_pUC	GTACATGTAGCGTTCAGGCGTAGAAAAGATCAAAGG	To amplify parts (Standard vector)
S2_pBR322	GTACATGTAGCGTTCAGGGGATTTGTTCAGAACGCT	To amplify parts (Standard vector)
S2_15A	GTACATGTAGCGTTCAGGGGATATATTCCGCTTCCT	To amplify parts (Standard vector)
S2_pSC101	GTACATGTAGCGTTCAGGGGCTTTTCTTGTATTATG	To amplify parts (Standard vector)
XthA.F	CGACTCTAGAGGATCATGAAATTTGTCTCTTT	Amplify *XthA* from Stellar genome
XthA.R	CGGTACCCGGGGATCTTAGCGGCGGAAGGTCG	Clone *XthA* from Stellar genome
chk_Nar1.1	CGGTTGGGAATGTAATTC	To verify Naringenin plasmid
chk_Nar1.2	CTCATGAGCGCTTGTTT	To verify Naringenin plasmid
chk_Nar1.3	ATTTCGGAGAAGGCGTAA	To verify Naringenin plasmid
chk_Nar1.4	CGGCTAACATCTTCTCAA	To verify Naringenin plasmid
chk_Nar1.5	AAGCAAGAGGTGACGAT	To verify Naringenin plasmid
chk_Nar1.6	ATAGTATCCTTTGGCTGG	To verify Naringenin plasmid
chk_Nar1.7	CCATTACCGAAGATGAAG	To verify Naringenin plasmid
chk_Nar1.8	TTATCTACACGACGGGGA	To verify Naringenin plasmid
chk_Nar1.9	CCTGAAGTAGTAGTCCGG	To verify Naringenin plasmid
chk_Nar1.10	TGCTCTTCATCCTCGAC	To verify Naringenin plasmid
chk_Nar1.11	CGTATAGGCGGTGTTGAG	To verify Naringenin plasmid
S1-J23119-R0034.F	CCTGAACGCTACATGTACTTGACAGCTAGCTCAGTCCTAGGTATAATGCTAGCTG TCTTGCTGTCTAGAGAAAGAGGAGAAATACTAG	88bp fragment
S1-J23119-R0034.R	CTAGTATTTCTCCTCTTTCTCTAGACAGCAAGACAGCTAGCATTATACCTAGGACT GAGCTAGCTGTCAAGTACATGTAGCGTTCAGG	88bp fragment
S1-J23106-R0034.F	CCTGAACGCTACATGTACTTTACGGCTAGCTCAGTCCTAGGTATAGTGCTAGCTG TCTTGCTGTCTAGAGAAAGAGGAGAAATACTAG	88bp fragment
S1-J23106-R0034.R	CTAGTATTTCTCCTCTTTCTCTAGACAGCAAGACAGCTAGCACTATACCTAGGACT GAGCTAGCCGTAAAGTACATGTAGCGTTCAGG	88bp fragment
J23101_R0034.R	CTAGTATTTCTCCTCTTTCTCTAGACAGCAAGACAGCTAGCATAATACCTAGGACT GAGCTAGCTGTAAAGATACCTTACCGCCGAAG	88bp fragment
J23101_R0034.F	CTTCGGCGGTAAGGTATCTTTACAGCTAGCTCAGTCCTAGGTATTATGCTAGCTG TCTTGCTGTCTAGAGAAAGAGGAGAAATACTAG	88bp fragment
J23100_R0034.F	CTAGGTTCTAACCGTCGATTGACGGCTAGCTCAGTCCTAGGTACAGTGCTAGCTG TCTTGCTGTCTAGAGAAAGAGGAGAAATACTAG	88bp fragment
J23100_R0034.R	CTAGTATTTCTCCTCTTTCTCTAGACAGCAAGACAGCTAGCACTGTACCTAGGACT GAGCTAGCCGTCAATCGACGGTTAGAACCTAG	88bp fragment
J23106_R0034.F	ACTCAGGAAGCAGACACTTTTACGGCTAGCTCAGTCCTAGGTATAGTGCTAGCTG TCTTGCTGTCTAGAGAAAGAGGAGAAATACTAG	88bp fragment
J23106_R0034.R	CTAGTATTTCTCCTCTTTCTCTAGACAGCAAGACAGCTAGCACTATACCTAGGACT GAGCTAGCCGTAAAAGTGTCTGCTTCCTGAGT	88bp fragment
J23119_B0034.1	TTGACAGCTAGCTCAGTCCTAGGTATAATGCTAGCTGTCTTGCT GTCTAGAGAAA	70bp fragment)overhang
J23119_B0034.2	CTAGTATTTCTCCTCTTTCTCTAGACAGCAAGACAGCTAGCATTATACCTAGGAC	70bp fragment-overhang
J23119_B0034.3	GTCCTAGGTATAATGCTAGCTGTCTTGCTGTCTAGAGAAAGAGGAGAAATACTA G	70bp fragment-overhang
J23119_B0034.4	TTTCTCTAGACAGCAAGACAGCTAGCATTATACCTAGGACTGAGCTAGCTGTCAA	70bp fragment-overhang
ho1_J23119.1	GGACTGAGCTAGCTGTCAATTTTTTTGACGGTAAAGCCA	Amplify backbone pho1
ho1_J23119.2	GAAAGAGGAGAAATACTAGGGTACCATGAGTGTCAACT	Amplify backbone pho1
bbho.1	GATCTTGATCCCCTGCG	Amplify backbone pho1
bbho.2	TGATCAAGAGACAGGATG	Amplify backbone pho1
prGFP70.F	TTGACAGCTAGCTCAGTCCTAGGTATAATGCTAGCTGTCTTGCTGTCTAGAGAAA GAGGAGAAATACTAG	70bp fragment
prGFP70.R	CTAGTATTTCTCCTCTTTCTCTAGACAGCAAGACAGCTAGCATTATACCTAGGACT GAGCTAGCTGTCAA	70bp fragment
60-119-34.F	TTGACAGCTAGCTCAGTCCTAGGTATAATGCTAGCTCTAGAGAAAGAGGAGAAA TACTAG	60bp fragment
60-119-34.R	CTAGTATTTCTCCTCTTTCTCTAGAGCTAGCATTATACCTAGGACTGAGCTAGCTG TCAA	60bp fragment
bb_dCas9.1	CACTGAAATCTAGAAATATTTTATCTGATTAATA	Amplify backbone pdCas9
bb_dCas9.2	TTTCTAGATTTCAGTGCCTAGGGATATATTAGTGCAA	Amplify backbone pdCas9

J23100_RBS.F	TTGACGGCTAGCTCAGTCCTAGGTACAGTGCTAGCAAGGAAGCTAAAGGAGGA<br>CAGAATT	60bp fragment
J23100_RBS.R	AATTCTGTCCTCCTTTAGCTTCCTTGCTAGCACTGTACCTAGGACTGAGCTAGCCG<br>TCAA	60bp fragment
bb_pNar_J23100.1	GACGGTTAGAACCTAGCTCGATCCTCTACGCCG	Amplify bb Naringenin plasmid
bb_pNar_J23106.1	TGTCTGCTTCCTGAGTCTCGATCCTCTACGCCG	Amplify bb Naringenin plasmid
bb_OsPKS_R0034.1	AGAGGAGAAATACTAGATGGCAGCGGCGGTGAC	Amplify bb Naringenin plasmid
MCoS.F	GAATTAAGGAGGACAGCTAA	Amplify bb Naringenin plasmid
OsPKS.R2	AGCTGTCCTCCTTAATTCAA	Amplify bb Naringenin plasmid
RSFori_Nar.1	TAGGCATGCAGCGCTCTT	Amplify bb Naringenin plasmid
RSFori_Nar.2	AAGAGCGCTGCATGCCTA	Amplify bb Naringenin plasmid
RSFori_Nar.3	ACTGGGTTGAAGGCTCTC	Amplify bb Naringenin plasmid
RSFori_Nar.4	GAGAGCCTTCAACCCAGT	Amplify bb Naringenin plasmid
3f_bb_dCas9	GGATATATTCCGCTTCCTCG	pdCas9 assembly
3f_dCas9_N1	CTATCGCCTTGTCCAGACACTTGTGCTTTTTGAAT	pdCas9 assembly
3f_dCas9_N2	CTAGGTTCTAACCGTCGATTGACGGCTAGCTCAG	pdCas9 assembly
3f_dCas9_C1	AAGCGGAATATATCCCTAG	pdCas9 assembly
3f_4k_bb_EL222.1	GTGAGCAAAAGGCCAGCA	4kb plasmid assembly
3f_4k_bb_EL222.2	AGTATGAAAAGTGACGTCG	4kb plasmid assembly
3f_4k_EL222.2	CGTCACTTTTCATACTCC	4kb plasmid assembly
3f_4k_EL222.1	CAATGTGGACTTGGAATTC	4kb plasmid assembly
3f_4k_EL222_RFP.2	TTCCAAGTCCACATTGAT	4kb plasmid assembly
3f_4k_EL222_RFP.1	CTGGCCTTTTGCTCACAT	4kb plasmid assembly
3f_5k_bb_EL222.1	ACGTCGGAATTGCCAGC	5kb plasmid assembly
3f_5k_bb_EL222.2	ACGGTTATCCACAGAATCA	5kb plasmid assembly
3f_5k_EL222.2	AATGTGGACTTGGAATTCAA	5kb plasmid assembly
3f_5k_EL222.1	CTGGCAATTCCGACGTC	5kb plasmid assembly
3f_5k_EL222_GFP.1	TTCTGTGGATAACCGTATTAC	5kb plasmid assembly
3f_5k_EL222_GFP.2	ATTCCAAGTCCACATTGAT	5kb plasmid assembly
PAL.F2	TATACCAGGACGTAACGAC	10kb plasmid assembly
4CL.F2	GATGCTCGCTTAGTGCTTA	10kb plasmid assembly
Nar_bb.F2	GGGTCTGACGCTCAGTGGA	10kb plasmid assembly
MCS.F2	TGAATTAAGGAGGACAGCT	10kb plasmid assembly
OsPKS.F2	GGAAGCAGCCCAGTAGTAG	10kb plasmid assembly
OsPKS.3	GATCCTGAAGTAGTAGTCC	10kb plasmid assembly
dCas9N.1	ATTTTTTTTGATACTGTGGC	6.3kb plasmid assembly
GFP.2	GAAAACTACCTGTTCCAT	3kb plasmid assembly
GFP.3	CATGGAACAGGTAGTTTTC	3kb plasmid assembly
GFP.4	TGGCAGACAAACAAAAGA	3kb plasmid assembly
GFP.5	TCTTTTGTTTGTCTGCCA	3kb plasmid assembly
Amp.1	AATGAAGCCATACCAAAC	3kb plasmid assembly
Amp.2	GTTTGGTATGGCTTCATT	3kb plasmid assembly
200S119-34.F	CCTGAACGCTACATGTACAAAATATTTCTAGCAAAAACCCCAGTTATTAAACCGC<br>CTAAGTCCCCCAGGAAAGGGGGATATAACAGTATAGATTTTGTCAGCCTTCAGCT<br>TGGCTTTACCGTCAAAAAAATTGACAGCTAGCTCAGTCCTAGGTATAATGCTAGC<br>TGTCTTGCTGTCTAGAGAAAGAGGAGAAATACTAG	200bp fragment
200S119-34.R	CTAGTATTTCTCCTCTTTCTCTAGACAGCAAGACAGCTAGCATTATACCTAGGACT<br>GAGCTAGCTGTCAATTTTTTTGACGGTAAAGCCAAGCTGAAGGCTGACAAAATCT<br>ATACTGTTATATCCCCCTTTCCTGGGGGACTTAGGCGGTTTAATAACTGGGGTTT<br>TTGCTAGAAATATTTTGTACATGTAGCGTTCAGG	200bp fragment
150S119-34.F	CCTGAACGCTACATGTACGAAAGGGGGATATAACAGTATAGATTTTGTCAGCCTT<br>CAGCTTGGCTTTACCGTCAAAAAAATTGACAGCTAGCTCAGTCCTAGGTATAATG<br>CTAGCTGTCTTGCTGTCTAGAGAAAGAGGAGAAATACTAG	150bp fragment
150S119-34.R	CTAGTATTTCTCCTCTTTCTCTAGACAGCAAGACAGCTAGCATTATACCTAGGACTGAGCTAGC<br>TGTCAATTTTTTTGACGGTAAAGCCAAGCTGAAGGCTGACAAAATCTATACTGTTATATCCCCC<br>TTTCGTACATGTAGCGTTCAGG	150bp fragment
100S119-34.F	CCTGAACGCTACATGTACAACACCCAATGTTTGACAGCTAGCTCAGTCCTAGGTATAATGCTA<br>GCTGTCTTGCTGTCTAGAGAAAGAGGAGAAATACTAG	100bp fragment

100S119-34.R	CTAGTATTTCTCCTCTTTCTCTAGACAGCAAGACAGCTAGCATTATACCTAGGACTGAGCTAGC<br>TGTCAAACATTGGGTGTTGTACATGTAGCGTTCAGG	100bp fragment
70S119-34.F	CTGAACGCTACATGTACTTGACAGCTAGCTCAGTCCTAGGTATAATGCTAGCAAAGAGGAGAA<br>ATACTAG	70bp fragment
70S119-34.R	CTAGTATTTCTCCTCTTTGCTAGCATTATACCTAGGACTGAGCTAGCTGTCAAGTACATGTAGC<br>GTTCAG	70bp fragment
RFP	atggcgagtagcgaagacgttatcaaagagttcatgcgtttcaaagttcgtatggaaggttccgttaacggtcacgagttcg<br>aaatcgaaggtgaaggtgaaggtcgtccgtacgaaggtacccagaccgctaaactgaaagttaccaaaggtggtccgctg<br>ccgttcgcttgggacatcctgtccccgcagttccagtacggttccaaagcttacgttaaacacccggctgacatcccggacta<br>cctgaaactgtccttcccggaaggtttcaaatgggaacgtgttatgaacttcgaagacggtggtgttgttaccgttacccagg<br>actcctccctgcaagacggtgagttcatctacaaagttaaactgcgtggtaccaacttcccgtccgacggtccggttatgcag<br>aaaaaaaccatgggttgggaagcttccaccgaacgtatgtacccggaagacggtgctctgaaaggtgaaatcaaaatgcg<br>tctgaaactgaaagacggtggtcactacgacgctgaagttaaaaccacctacatggctaaaaaaccggttcagctgccggg<br>tgcttacaaaaccgacatcaaactggacatcacctcccacaacgaagactacaccatcgttgaacagtacgaacgtgctga<br>aggtcgtcactccaccggtgcttaa	
GFP	atgcgtaaaggagaagaacttttcactggagttgtcccaattcttgttgaattagatggtgatgttaatgggcacaaattttct<br>gtcagtggagagggtgaaggtgatgcaacatacggaaaacttacccttaaatttatttgcactactggaaaactacctgttc<br>catggccaacacttgtcactactttcggttatggtgttcaatgctttgcgagatacccagatcatatgaaacagcatgactttt<br>tcaagagtgccatgcccgaaggttatgtacaggaaagaactatatttttcaaagatgacgggaactacaagacacgtgctg<br>aagtcaagtttgaaggtgatacccttgttaatagaatcgagttaaaaggtattgattttaaagaagatggaaacattcttgg<br>acacaaattggaatacaactataactcacacaatgtatacatcatggcagacaaacaaaagaatggaatcaaagttaact<br>tcaaaattagacacaacattgaagatggaagcgttcaactagcagaccattatcaacaaaatactccaattggcgatggcc<br>ctgtccttttaccagacaaccattacctgtccacacaatctgccctttcgaaagatcccaacgaaaagagagaccacatggt<br>ccttcttgagtttgtaacagctgctgggattacacatggcatggatgaactatacaaataa	
sfGFP	atgcgtaaaggcgaagagctgttcactggtgtcgtccctattctggtggaactggatggtgatgtcaacggtcataagttttc<br>cgtgcgtggcgagggtgaaggtgacgcaactaatggtaaactgacgctgaagttcatctgtactactggtaaactgccggt<br>accttggccgactctggtaacgacgctgacttatggtgttcagtgctttgctcgttatccggaccatatgaagcagcatgactt<br>cttcaagtccgccatgccggaaggctatgtgcaggaacgcacgatttcctttaaggatgacggcacgtacaaaacgcgtgc<br>ggaagtgaaatttgaaggcgataccctggtaaaccgcattgagctgaaaggcattgactttaaagaagacggcaatatcct<br>gggccataagctggaatacaattttaacagccacaatgtttacatcaccgccgataaacaaaaaaatggcattaaagcga<br>attttaaaattcgccacaacgtggaggatggcagcgtgcagctggctgatcactaccagcaaaacactccaatcggtgatg<br>gtcctgttctgctgccagacaatcactatctgagcacgcaaagcgttctgtctaaagatccgaacgagaaacgcgatcatat<br>ggttctgctggagttcgtaaccgcagcgggcatcacgcatggtatggatgaactgtacaaatga	

Table S3. Summary of sequenced colonies

Short-frag	Size (bp)	Template	Success rate	Construct ref
J23106-34	88	GFP (2.8kb)	2/3	S7b
J23119-34	88	GFP (2.8kb)	2/3	S7c
J23101-34	88	GFP (2.8kb)	1/1	S7a
J23101-34	88	sfGFP (2.8kb)	3/3	S7d
J23101-34	88	sfGFP (4.2kb)	3/3	S7e
J23100-34	88	dCas9 (6.3kb)	3/3	S7g
J23106-34	88	pNar (10.3kb)	2/2	S7f
J23119-34	70	pho1 (3.0kb)	12/12	S7h

Cropped figures	Original gel figures

Figure 1	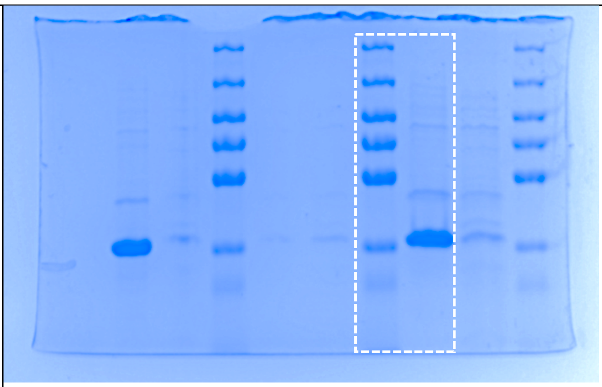
Figure 2	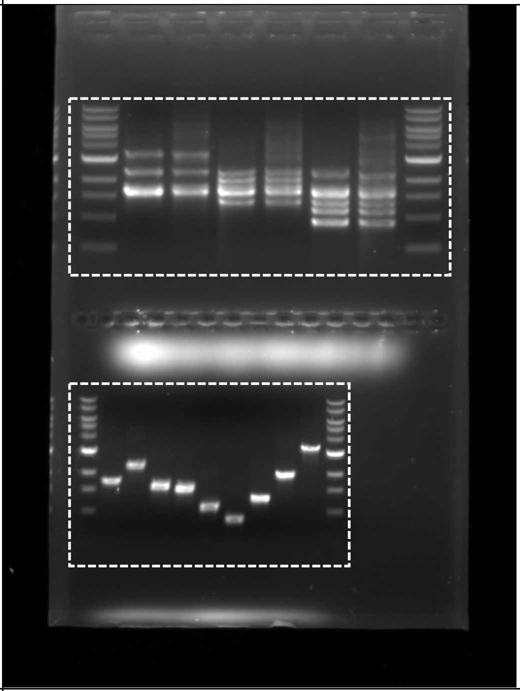
Figure 3a	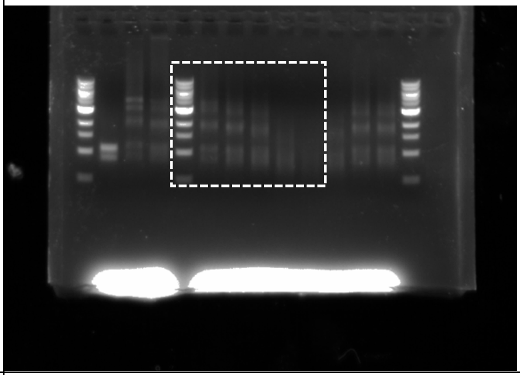
Figure 3b	

	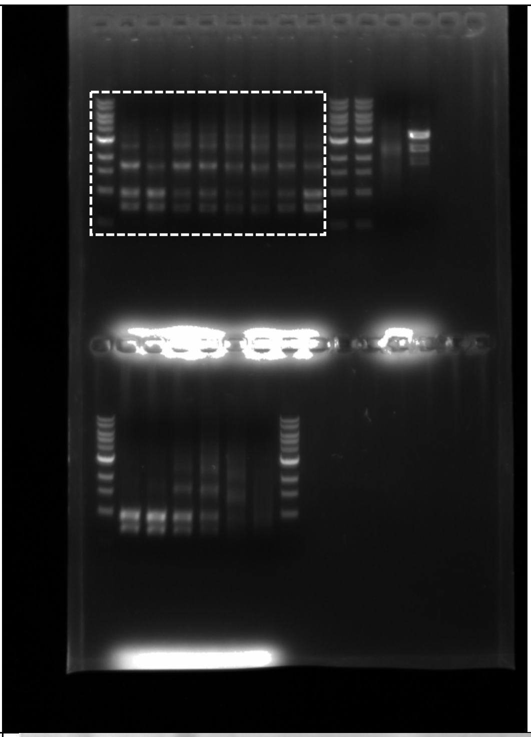
Figure S4a & S4b	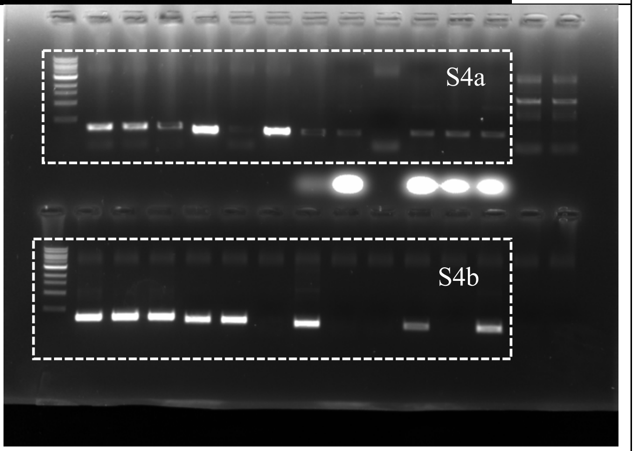
Figure S4a	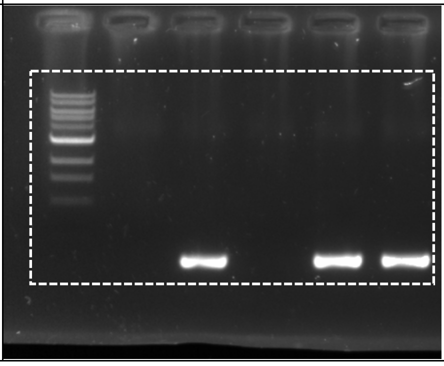

Figure S9a & S4b	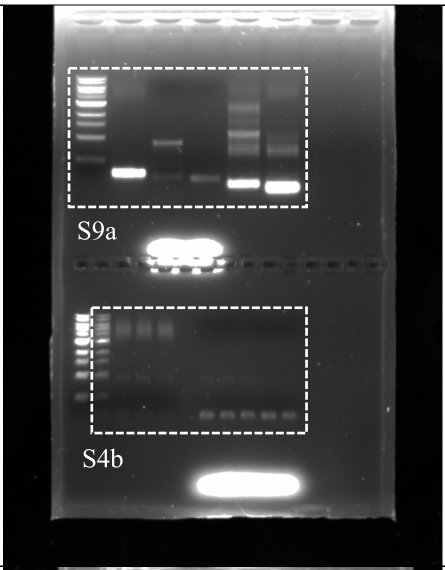	
Figure S9b	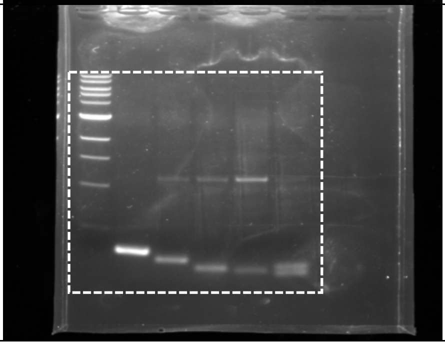	

Table S4. Obtained colony number

Experiments	1st	2nd	3rd	Figure
Sample's name	Number of colonies			
Control	0	0	0	Fig. 1a
10Beta-NEB	30	51	35	
5Alpha-NEB	150	252	187	
Stellar-Takara	86	215	105	
Control (A)	0	0	0	Fig. 1b
SENAX (A)	192	145	115	
Ctl (B)	1	3	4	
SENAX (B)	170	150	90	
Ctl (C)	0	0	1	
SENAX (C)	18	54	41	
Ctl (D)	0	0	1	
SENAX (D)	18	54	41	
Ctl (E) (4.0kb)	2	0	0	
SENAX (4.0kb)	17	29	12	
Ctl (F) (5.0kb)	1	1	0	
SENAX (5.0kb)	62	90	45	

Ctl (G) (6.3kb)	2	1	1	
SENAX (6.3kb)	95	100	79	
Ctrl-3	1	0	0	Fig. 2a
3-frags	230	169	112	
Ctrl-4	0	0	0	
4-frags	55	46	22	
Ctrl-5	0	0	0	
5-frags	47	32	19	
Ctrl-6	0	0	0	
6-frags	12	17	9	
Control-3	2	2	3	Fig. 2b
3-frags	151	286	301	
Control-4	2	2	1	
4-frags	22	37	17	
Control-5	1	0	0	
5-frags	8	4	5	
Control-6	1	0	0	
6-frags	3	1	2	
Control-7	0	0	1	
7-frags	2	1	3	
SENAX-200bp	59	99	77	Fig. 3a
In-Fusion-200bp	40	65	48	
Gibson-200bp	11	16	1	
SENAX-150bp	22	35	34	
In-Fusion-150bp	39	32	34	
Gibson-150bp	29	24	21	
SENAX-100bp	11	11	13	
In-Fusion-100bp	3	7	2	
Gibson-100bp	1	1	0	
SENAX-88bp	38	16	25	
In-Fusion-88bp	3	1	3	
Gibson-88bp	0	0	0	
SENAX-70bp	14	8	8	
In-Fusion-70bp	5	1	2	
Gibson-70bp	0	0	0	
SENAX-200bp	35	20	29	Fig. 3b
In-Fusion-200bp	14	28	18	
Gibson-200bp	1	0	0	
SENAX-150bp	27	16	22	
In-Fusion-150bp	14	27	21	
Gibson-150bp	2	6	2	
SENAX-100bp	10	15	10	

In-Fusion-100bp	4	9	4	
Gibson-100bp	0	0	0	
SENAX-88bp	11	14	12	
In-Fusion-88bp	2	4	2	
Gibson-88bp	0	0	0	
SENAX-70bp	19	12	17	
In-Fusion-70bp	3	1	4	
Gibson-70bp	0	0	0	
SENAX-200bp	13	8	4	Fig. 3c
In-Fusion-200bp	1	1	1	
Gibson-200bp	1	1	0	
SENAX-150bp	2	5	4	
In-Fusion-150bp	1	2	2	
Gibson-150bp	1	1	0	
SENAX-100bp	3	7	5	
In-Fusion-100bp	0	0	0	
Gibson-100bp	0	0	0	
SENAX-88bp	5	7	2	
In-Fusion-88bp	1	0	0	
Gibson-88bp	0	0	0	
SENAX-70bp	6	2	4	
In-Fusion-70bp	1	0	0	
Gibson-70bp	0	0	0	
0ng	0	0	0	Fig. 4a
10ng	178	251	254	
20ng	253	291	231	
30ng	302	158	181	
50ng	26	20	31	
100ng	0	0	0	
25°C	40	9	11	Fig. 4b
28°C	30	10	10	
30°C	87	48	75	
32°C	118	57	85	
35°C	101	52	67	
37°C	72	49	89	
42°C	80	50	90	
50°C	11	5	34	
0min	3	7	4	Fig. 4c
5min	52	59	74	
10min	138	129	111	
15min	113	120	172	
20min	150	77	118	

30min	77	168	106	
60min	25	67	31	
0mM	13	5	10	Fig. 4d
20mM	15	12	18	
50mM	40	26	22	
100mM (+dNTPs)	70	61	37	
100mM	75	55	45	
200mM	105	48	56	
300mM	128	72	68	
500mM	80	50	28	
4-bp 5'-overhang	90	63	78	Fig. S8a
4-bp 3'-overhang	0	1	0	
Blunt-end	105	71	122	
18bp	1125	1053	-	Fig. S10
15bp	790	578	-	
12bp	194	195	-	
10bp	8	22	-	
